# A Systematic Review of New, Enhanced Surveillance Systems and Methodologies for Zoonotic Influenza Viruses in Animals and Human–Animal Interface

**DOI:** 10.1111/irv.70178

**Published:** 2025-11-10

**Authors:** Rebecca Badra, Wenqing Zhang, John S. L. Tam, Richard Webby, Sylvie Van Der Werf, Sergejs Nikisins, Ann Cullinane, Saad Gharaibeh, Richard Njouom, Malik Peiris, Ghazi Kayali, Jean‐Michel Heraud

**Affiliations:** ^1^ Human Link DMCC Dubai UAE; ^2^ World Health Organization HQ/WPE/EPP/GIP Geneva Switzerland; ^3^ The Hong Kong Polytechnic University Hong Kong Hong Kong SAR China; ^4^ St. Jude Children's Research Hospital Memphis Tennessee USA; ^5^ Molecular Genetics of RNA Viruses Unit, Institut Pasteur Paris France; ^6^ The Irish Equine Centre, Johnstown, Naas Ireland; ^7^ Jordan University of Science and Technology Irbid Jordan; ^8^ Centre Pasteur du Cameroun Yaoundé Cameroon; ^9^ School of Public Health The University of Hong Kong Hong Kong Hong Kong SAR China; ^10^ Virology Unit Institut Pasteur de Madagascar Antananarivo Madagascar

**Keywords:** domestic animals, surveillance, surveillance system, wildlife, zoonotic influenza virus

## Abstract

In 2009, the World Health Organization (WHO) developed a public health research agenda for influenza to guide researchers and outline directions and priority areas for research on influenza aiming at reducing the burden of seasonal epidemic influenza and the risk and impact of pandemic influenza. The agenda was updated in 2017, but since then, important research has been conducted, and major changes have occurred to the global health landscape impacted mainly by the COVID‐19 pandemic. Therefore, there is a need to assess advances in zoonotic influenza surveillance methods reported between 2017 and 2024 in order to highlight key achievements and identify remaining gaps that limit their broader implementation, hence informing an update of the research agenda. We conducted a comprehensive literature review of zoonotic influenza surveillance and monitoring, focusing on novel and enhanced methodologies reported globally between 2017 and 2024. A systematic analysis was performed following PRISMA guidelines on 7490 peer‐reviewed manuscripts from 2017 to 2024 retrieved from PubMed, of which 164 records were included in this review. Analysis of the information collected indicated several advances and gaps at different levels of surveillance and unmet public health needs. Most countries do not have active and comprehensive surveillance programs for zoonotic influenza at the human–animal interface, which underestimates the true burden of zoonotic influenza diseases. The review concludes with a set of high‐priority research recommendations focused on filling gaps in One Health data integration, validation, and field deployment of novel diagnostic technologies, wider adoption of noninvasive and environmental surveillance approaches, and stronger linkage of methodological innovations to risk assessment and policy action. In light of the recent upsurge in H5N1 activity and cross‐species transmission, the WHO has convened multiple R&D Blueprint consultations over the past year to prioritize research and development for H5N1 candidate vaccines, diagnostics, and pandemic preparedness. These ongoing initiatives underscore the critical importance of strengthening surveillance at the human–animal interface.

## 1 Introduction

Zoonotic influenza viruses pose a major public health concern due to their capacity to cross the species barrier and infect humans, often with devastating consequences [1]. The 2009 pandemic due to A(H1N1)pdm09, showed how zoonotic viruses can rapidly adapt to infect humans with a virus likely originating from swine and harboring genetic components from human, swine, and avian influenza A viruses [2]. Within months of its initial detection on a pig farm in Mexico, the virus spread globally, resulting in millions of infections and substantial mortality, particularly among vulnerable populations such as young children and those with underlying health conditions [3]. The rapid transmission of A(H1N1)pdm09 was facilitated by several factors, including the interconnectedness of global travel and trade, which allowed the virus to transmit across borders and continents with alarming speed [4]. Public health systems worldwide were overwhelmed and struggled to keep pace with the outbreak's dynamics [5] highlighting weaknesses in existing surveillance programs and the need for robust surveillance systems that can effectively monitor and detect influenza in both wildlife and domestic animals that could serve as reservoirs for emerging pathogens. Moreover, the A(H1N1)pdm09 experience highlighted the importance of interspecies transmission pathways, as the virus was linked to pigs and other animals before it adapted to human hosts [6]. This emphasized the need for a One Health approach that recognizes the interconnectedness of human, animal, and environmental health. The pandemic underscored the critical role of surveillance systems in monitoring human health outcomes but also in tracking influenza activity in animal populations, enabling early detection of potential spillover events. The complex ecology of influenza viruses in wildlife and domestic animals further complicates detection efforts [7]. In recent years, the emergence of novel influenza strains, driven by factors such as climate change, habitat destruction, and increased human–animal interactions has facilitated the exchange of pathogens and increased the risk of zoonotic transmission [8]. The recent outbreaks of highly pathogenic avian influenza (HPAI) H5N1 in cattle and goats in the United States of America (US), the detection of the virus in deceased cats infected likely through unpasteurized milk from infected cows, and reported infections in farm workers raise serious global health concerns, highlighting the virus's expanding host range and zoonotic potential [9, 10, 11]. This dynamic underscores the necessity for comprehensive surveillance strategies that can monitor these interactions in real‐time.

To enhance global knowledge and response to influenza, the World Health Organization (WHO)’s Global Influenza Programme developed the WHO public health research agenda for influenza in 2009. This agenda identifies research priorities for pandemic, zoonotic, and seasonal influenza, emphasizing a multidisciplinary approach and collaboration among researchers, public health officials, and policymakers. It was reviewed in 2010–2011 and 2017 to guide research for the next 5–10 years. Since 2017, significant research and changes, especially due to COVID‐19, have occurred, necessitating an assessment of knowledge and data gaps from 2017 to 2024. The unprecedented spread of HPAI H5N1 among wild birds, domestic animals, cattle and recent human cases have raised serious global health concerns. In response, WHO convened an R&D Blueprint consultation to prioritize containment and mitigation measures of pandemic H5N1 influenza [12]. Integrating such global R&D efforts with improved field surveillance will be crucial for pandemic preparedness.

In this study, we conducted a comprehensive literature review to systematically assess novel and enhanced surveillance systems and methods for zoonotic influenza viruses published between 2017 and 2024. This focus directly responds to the 2017 WHO Public Health Research Agenda for Influenza, which prioritized improvements in surveillance tools and approaches at the human–animal interface. We do not aim to cover all aspects of zoonotic influenza research in this review; instead, we highlight advances in surveillance systems and methodologies, summarize their reported effectiveness (including sensitivity, specificity and timeliness), and identify remaining limitations and gaps that affect the broader status of zoonotic influenza surveillance. By aligning recent innovations with the WHO research priorities, this review provides a clearer picture of progress made and areas still requiring attention. The review focuses on enhanced and novel reported surveillance systems that are in place for outbreaks of influenza A viruses (IAVs) in wild and domestic animals highlighting application of surveillance in wild animals, the environment, domestic animals, and exposed humans. It also highlights novel methodologies and approaches and assesses their effectiveness in terms of reported performance metrics such as sensitivity, specificity, timeliness, feasibility for field deployment, and contribution to improved risk assessment of zoonotic IAVs.

## 2 Methods

### 2.1 Search Strategy

**FIGURE 1:**
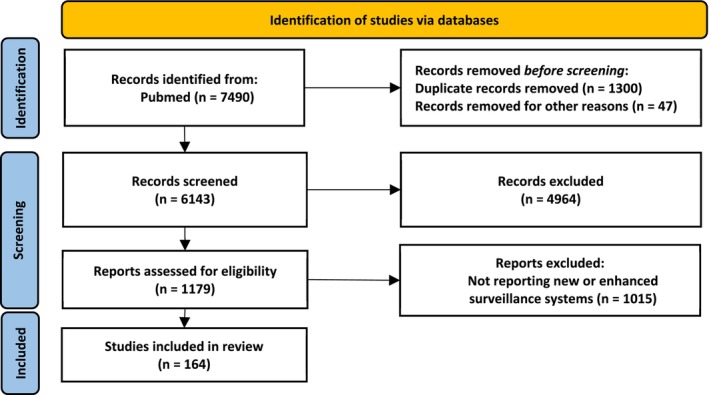
PRISMA flow diagram of the screening and selection of studies for this review.

This study was designed following the Preferred Reporting Items for Systematic Reviews and Meta‐Analyses (PRISMA) 2020 for review of the published peer‐reviewed literature. This study did not require institutional review board approval. We used PubMed to search for English‐language peer‐reviewed publications using the search terms “avian influenza,” “swine influenza,” and “zoonotic influenza viruses” from 2017 till 2024 (Figure 1). In total, 7490 records were exported to an Endnote X8 (Endnote, Berkley, CA) library (Figure 1). Duplicates and papers published after 31 March 2024 were removed, yielding 6143 publications retained and exported to a master Excel spreadsheet (Microsoft, Redmond, WA). The following data were extracted from each included study: publication year, country, and category. We then searched for the keyword “surveillance” in the master Excel spreadsheet to select the papers that discussed surveillance. The search yielded 1179 manuscripts. Those records were exported to a new Excel sheet. Titles and abstracts were reviewed thoroughly and were independently screened by two researchers to select the papers discussing new and enhanced methodologies and surveillance systems for detection of zoonotic influenza viruses. Additional records from the initial library (6143), which did not include the word “surveillance” but showed to be relevant, were re‐included to ensure that all relevant publications were captured. A total of 164 research papers were included in this review. The selection was confirmed by a senior scientist with extensive expertise in public health and more than 10 years of experience in zoonotic influenza.

### 2.2 Eligibility Criteria

The included publications met the following eligibility criteria: (1) publications reporting new or enhanced methodologies or surveillance systems for detection of zoonotic influenza viruses; (2) surveillance conducted in at least one of the following contexts: domestic animal husbandry (farmed poultry, swine, cattle, goats, and related production systems, including live bird markets and smallholder farms), wild animals (e.g., wild birds or other wildlife reservoirs), environmental samples (e.g., air, water, wastewater, soil), or exposed humans at the human–animal interface; (3) publications are published in the period from 2017 to the end of March 2024. Publications were excluded for the following reasons: (1) duplicates, (2) published after March 31, 2024, (3) records not reporting new or enhanced surveillance systems for detection of zoonotic influenza viruses.

### 2.3 Data Collection Process

We collected data from the 164 retained peer‐reviewed publications related to enhanced and novel surveillance systems for zoonotic influenza virus applied in domestic animal husbandry (farmed poultry, swine, cattle, goats, and related production systems) and exposed humans, enhanced and novel surveillance systems applied in animals in the wild, and enhanced and novel surveillance systems applied to the environment (air, water, wastewater). We also extracted information related to novel methodologies and approaches for detection and forecasting. Additionally, we extracted any information related to assessing and studying the effectiveness of the enhanced or novel surveillance systems in improving risk assessment on human infection by animal IAVs.

## 3 Results

### 3.1 Study Selection

A total of 1179 records whose title and/or abstract contain the keyword “surveillance” were retained from PubMed and were thoroughly screened to extract information related to new and enhanced methodologies and surveillance systems for detection of zoonotic influenza viruses. A total of 164 peer‐reviewed papers were included in this review (Figure 1). Most studies originated from East and Southeast Asia (e.g., China, Japan, Republic of Korea, Thailand), Europe (e.g., Italy, United Kingdom (UK), Netherlands, France, Germany, Denmark), and North America (primarily US and Canada). Fewer studies were conducted in Africa (e.g., South Africa), South America (e.g., Brazil, Argentina), and the Middle East (e.g., Iran).

### 3.2 Findings

Since 2017, several advances in developing novel influenza surveillance systems or enhancing existing surveillance programs to improve monitoring of influenza viruses and enhance early detection and response were reported. Livestock and poultry are susceptible to IAVs and should be monitored closely when outbreaks in wildlife are detected and through routine surveillance. The application of a One Health approach is crucial to detect emerging influenza zoonotic viruses that can pose a threat to exposed humans.

#### 3.2.1 Application in Domestic Animal Husbandry and Exposed Humans

##### 3.2.1.1 Predictive and Risk‐Based Surveillance Systems

Over the past 8 years, predictive surveillance systems have been developed to assess the risk of zoonotic influenza transmission from wild to domestic animals using influenza transmission, demographic, and spatial risk factors. The RealOpt‐ASSURE system effectively integrates compartmental modelling with human behavior data for monitoring avian influenza virus (AIVs) outbreaks [13]. Research has shown that analyzing the density of avian influenza‐infected dabbling ducks in Northern Italy could be used to identify the risk of introduction of AIVs into poultry flocks [14]. Avian influenza‐driven risk maps have been created to evaluate poultry and wild bird densities in England for targeted risk‐based sampling strategies [15]. Studies also link wild bird densities to the risk of HPAI infections in poultry in the Netherlands [16]. The Random Forest model has been identified as the most effective predictive model for estimating IAVs frequency in swine in Ontario, Canada [17]. A machine learning framework has been proposed for rapid HPAI risk prediction, considering environmental and meteorological data, farm biosecurity measures, and wild bird HPAI surveillance data [18]. A predictive framework utilizing an extensive spatiotemporal dataset has been developed to understand AIVs drivers and forecast future outbreaks in Korea [19].

##### 3.2.1.2 Community‐Based and Digital Surveillance Approaches

In Kenya, mobile phone syndromic surveillance has been useful for detecting severe diseases but may not catch early avian influenza outbreaks [20]. A participatory One Health disease detection system was developed in Thailand as a novel community‐owned disease surveillance system that allows community members to monitor health issues, facilitating early disease detection [21]. An integrated behavioral biological surveillance approach that targets high‐risk populations where humans, livestock, and wildlife interact was applied in Thailand, helping policymakers enhance surveillance strategies and behavior‐focused interventions [22]. Additionally, a study in the UK analyzed posts on the social media platform Twitter (now X) and found only a weak correlation between avian influenza–related messages and confirmed cases, suggesting that social media data alone may have limited reliability for surveillance but could provide supplementary insights when combined with traditional systems [23].

##### 3.2.1.3 Enhanced Sampling Strategies

Enhanced sampling approaches were reported from 2017 to 2024. A study comparing individual, group, and environmental sampling methods for influenza surveillance in pigs in the US found that group and environmental sampling were more effective for detection, while individual sampling is better for viral isolation and sequencing [24]. Udder skin wipes from lactating sows were identified as a cost‐effective and sensitive method for detecting and isolating IAVs in the US, with pooling up to three wipes not affecting sensitivity [25]. In swine farm settings, two studies evaluated bioaerosol and surface sampling as early detection tools, emphasizing the importance of sampling environment design on detection outcomes [26, 27]. Another study further demonstrated that using additives such as bovine serum albumin (BSA) in environmental dust samples can significantly improve real‐time quantitative reverse‐transcriptase polymerase chain reaction (RT‐qPCR) sensitivity [28]. Although few studies explicitly address the use of sentinel birds in large poultry units, targeted wild bird surveillance approaches suggest parallels that could inform sentinel‐based strategies [29, 30]. Monitoring urban rats was effective for studying zoonotic viruses in Vienna, Austria [31]. Another study showed that combining active surveillance in commercial game birds with mortality‐based triggers improved HPAI detection rates [32]. Duck hunting preserves in Maryland, US were identified as a useful model for studying AIV dynamics among nonmigratory birds [33]. A study showed the feasibility of asymptomatic avian influenza surveillance in exposed persons in England as an approach for avian influenza detection [34]. Lastly, innovative methods, including sediment sampling from wetlands in Canada [35] and a virion enrichment technique developed in Japan (MiVET) [36], improved detection of AIVs in various environments.

##### 3.2.1.4 Integrated One Health Surveillance Systems

Thailand piloted a One Health avian influenza surveillance system in 2016, integrating human, animal, and environmental stakeholders to monitor influenza A viruses, which involves triggering public health measures upon suspected cases, coordinating sample collection and testing, and sharing results while highlighting both strengths in simplicity and flexibility and challenges in data quality and interoperability [37].

##### 3.2.1.5 Data Visualization and Analytical Tools

Tools for surveillance data visualization were developed to improve access to and interpretation of influenza surveillance data. The Iowa State University veterinary diagnostic laboratory developed a website for visualizing real‐time swine IAV data and linking it to the United States Department of Agriculture (USDA) surveillance system [38]. In addition, OctoFLUshow, an interactive platform, was developed to analyze and report IAV strains collected since 2009. A complementary study presented a systematic approach for analyzing USDA swine IAV surveillance sequencing data, integrated with OctoFLUshow to provide a searchable overview of strain diversity in the US [39].

#### 3.2.2 Application in Animals From the Wild

Surveillance efforts for AIVs have begun to expand beyond traditional domestic and avian hosts to include less commonly monitored species. A study explored urban zoonotic virus surveillance using city rats, and while not focused on canids, the study highlights the potential of opportunistic surveillance in urban wildlife [31]. Predictive systems for surveillance of avian influenza in the wild were developed. A gradient boosted trees method enhanced active surveillance for AIVs in the US using geographic location and RT‐qPCR results as predictors [40]. The Observation.org platform effectively monitored wildlife pathogens by correlating observations of sick and dead birds with outbreaks reported by the World Organisation for Animal Health (WOAH), aiding early detection of HPAI outbreaks in Belgium and the Netherlands [41]. Additionally, the Citizen Scientist eBird database helped map wild bird distributions in Taiwan, informing cost‐effective AIV surveillance site selections [42]. A spatiotemporal model based on habitat suitability and avian influenza detection coordinates was developed to support a risk‐based surveillance system for HPAI prevention and preparedness [43]. Three active sampling methods showed that hunter‐sourced waterfowl sampling in the UK could detect avian influenza before the occurrence of outbreaks on poultry, allowing for early intervention to protect national poultry [29]. A new method for sequencing hemagglutinin and neuraminidase directly from wild bird faucal RNA using Nanopore Flongle proved to be cost‐effective and easier than traditional methods [44]. Additionally, a “fireworks” spatiotemporal model was developed to simulate and visualize the spread of HPAI cases over time and across geographic areas, named for the way outbreaks radiate outward like fireworks, thereby supporting the improvement of surveillance systems [45].

#### 3.2.3 Application to the Environment

From 2017 to 2024, novel environmental sampling methods have been developed for avian influenza detection and are summarized in Table 1. An approach has been designed to improve the detection of AIV in California wetlands utilizing remote sensing, filtration, and sequencing [46]. Bioaerosol and surface sampling showed effectiveness in swine surveillance in Canada, complemented by a mobile data collection application [26]. A noninvasive bioaerosol surveillance strategy was tested for detecting swine pathogens in North Carolina swine farms [27]. The addition of BSA enhanced RT‐qPCR sensitivity for HPAI virus detection in dust samples from French poultry farms affected by A(H5N1) outbreaks [28]. A study conducted in South Africa showed that fresh faucal samples collected immediately have detection rates similar to or higher than oropharyngeal and cloacal swabs [30]. Other methods included using feather samples for noninvasive detection in commercial poultry in the US [49] and combining hydrodynamic models with mallard abundance data to identify AIV hotspots in Southern France [47]. A spatial model was developed in Argentina to identify high‐risk areas for low pathogenic avian influenza virus occurrence at the backyard poultry‐wild bird interface using expert opinions and ecological niche modelling to inform surveillance [50]. A spatial framework assessed regions at high risk for influenza virus spillover by quantifying the geographic variation in outbreak emergence potential [51]. Additionally, two water sampling methods were developed in the Netherlands for AIV detection, with the 1 L method proving quicker and easier to process compared to the 50 L method, both effective for environmental screening [48]. A review suggested that environmental sampling may not be a reliable detection method especially when the infection and contamination are less prevalent, and thus information about the species and source of the virus might be missed [52].

**TABLE 1:** Novel environmental sampling methods developed globally from 2017 to 2024.

Environmental sample	Sampling technique	Aim	Uses for surveillance
Water	Combining remote sensing, filtration and sequencing [46]	Improving environmental surveillance	Novel and effective approach for AIV monitoring
Water	Combining a hydrodynamic model with data on mallard abundance and AIV infection rate [47]	Creating AIV transmission risk maps	Efficient for improved surveillance
Water	1 L and 50 L water sampling [48]	Detection of AIV in water	Detection of AIV in wetlands
Bioaerosol	Swine oral fluids, surface swabs, and air sampling [26]	Detection of influenza virus in swine	Effective noninvasive approach for detecting influenza virus among swine
Bioaerosol	Air sampling [27]	Detection of swine pathogens	Effective noninvasive approach for detecting pathogens in animals
Dust	Dry wipes rubbed against the walls or feeders [28]	Viral surveillance with the addition of BSA to eliminate inhibitors	Effective method for HPAI monitoring
Feces	Fresh feces sampling (droppings) [30]	Pathogen surveillance in wild birds	Cost‐effective, rapid, and noninvasive method that can be applied under sub‐Saharan African conditions
Feathers	Contour feather sampling [49]	AIV surveillance in birds	Effective noninvasive method for the accurate detection of AIV in commercial poultry

#### 3.2.4 Novel Methodologies and Approaches for Detection and Forecasting

Conventional surveillance methods have been improved by the adoption of real‐time PCR and the upgrading and designing of new methods and assays for the rapid detection and characterization of influenza viruses. Furthermore, advancements in next‐generation sequencing technology and the emergence of genomic surveillance approaches allowed the genetic analysis of viral strains and understanding of their origin and spillover potential.

##### 3.2.4.1 Nucleic Acid Amplification–Based Methods

New reported nucleic acid amplification methods for detecting AIVs included a rapid convection PCR (cPCR) platform for A(H5) and A(H9) subtypes [53], and an isothermal reverse transcription recombinase polymerase amplification with lateral‐flow dipstick (RT‐RPA‐LFD) assay that is 10 times more sensitive than conventional RT‐PCR for A(H9) detection [54]. A lateral flow dipstick recombinase polymerase amplification (LFD‐RPA) assay achieved 100% sensitivity and specificity for A(H7N9) without cross‐reactivity [55]. Additional methods like real‐time fluorescence and reverse transcription recombinase‐aided amplification (RF‐RT‐RAA), reverse transcription recombinase‐aided amplification combined with lateral flow dipstick (RT‐RAA‐LFD) [56, 57], and a CRISPR‐Cas13a based platform were developed for rapid detection of various avian influenza subtypes [58]. Real‐time PCR methods including four singleplex RT‐qPCR assays were developed for the rapid and simultaneous detection of avian influenza subtypes A(H4), A(H6), and A(H10), suitable for laboratory and clinical use [59]. A USDA‐validated RT‐qPCR targeting the M gene effectively detects most influenza A viruses and adapts to new strains [60]. Additional assays include a real‐time probe‐based assay for the rapid and specific detection of endemic A(H9N2) AIV [61], a TaqMan RT‐qPCR for A(H2) [62], a sensitive Uni Kor‐H9 assay for various A(H9Nx) viruses [63], a TaqMan RT‐qPCR for A(H5) [64], and a cost‐effective highly pathogenic A(H5) RT‐qPCR for clade 2.3.4.4b A(H5) virus detection [65]. Novel multiplex PCR methods [66, 67, 68, 69, 70, 71, 72, 73, 74, 75, 76, 77, 78, 79, 80, 81, 82, 83, 84, 85, 86, 87, 88, 89, 90, 91, 92, 93, 94, 95, 96] developed from 2017 to 2024 are summarized in Table 2.

**TABLE 2:** Multiplex PCR methods developed from 2017 to 2024 for the detection of influenza viruses.

Method	Type detected	Study country
Multiplex RT‐qPCR [66]	Detection of AIVs subtypes H5, H7, and H9	China
Tetraplex RT‐qPCR [67]	Simultaneous screening of four influenza virus types, A, B, C and D in swine	Germany
Triplex RT‐qPCR [68]	Detection of duck‐origin AIVs, Newcastle disease virus (NDV), and duck Tembusu virus (DTMUV)	China
Triplex RT‐qPCR [69]	Simultaneous detection of AIVs subtypes H3, H4, and H5	China
Triplex RT‐qPCR [70]	Simultaneous detection of AIVs subtypes H5, H7, and H9	China
Multiplex RT‐qPCR assay using TaqMan minor groove binder (MGB) probes [71]	Detection of H10 subtype AIVs	China
Multiplex RT‐qPCR [72]	Simultaneously detection of pan‐H5 HPAI viruses and H5Nx clades 2.3.2.1 and 2.3.4.4 viruses	Republic of Korea
Quadruple RT‐qPCR [73]	Simultaneous detection of H7N9 and identification of HP and NAI‐resistance mutations	China
Double RT‐qPCR [74]	Detection of H5N6 influenza virus	China
Multiplex one‐step RT‐qPCR [75]	Detection of nine AIVs NA subtype	China
Multiplex RT‐qPCR [76]	Detection of H6 AIVs	China
RT‐PCR assays using newly designed NS and PB2 primers and probes [77]	Detection of IAVs	China
TaqMan probe chemistry (TaqMan multitarget) [78]	Simultaneous detection of AIVs (M gene) and subtyping (H5, N1, H9, N2)	Bangladesh
RT‐qPCR [79]	Detection of two H9 lineages of AIVs	Japan
RT‐qPCR [80]	Detection of the novel H5N6 virus	China
Real‐time RT‐PCR [81]	Detection of Cluster IV H3N2v	Japan
SYBR(R) Green‐based RT‐qPCR [82]	Subtyping of avian influenza HA	US
Multiplex RT‐PCR [83]	Detection and subtyping of IAVs	Brazil
Multiplex RT‐qPCR [84]	Detection of H5 AIVs	China
GeXP multiplex RT‐PCR [85]	Detection of eight AIVs subtypes (H1, H2, H3, H5, H6, H7, H9 and H10)	China
Multiplex RT‐qPCR [86]	Differentiation of the NA and HA genes of the three major IAVs subtypes	Brazil
qPCR assays [87]	Targeting of HA and NA gene lineages relevant for swIAV and detection of internal genes of IAV	Denmark
Pan‐AIV RT‐qPCR [88]	Detection of all AIVs	China
H5‐AIV RT‐qPCR [88]	Detection of H5 AIVs	China
Pan‐H9 RT‐qPCR [89]	Detection H9Nx viruses of any of the Y439, Y280, and G1 clades	Italy
RT‐qPCRs [90]	Subtyping of European swine IAVs	France
Multianalyte suspension assay (MASA) combining one‐tube multiplex RT‐qPCR with bead hybridization and detection [91]	Subtyping of AIVs	China
Real‐time reverse transcription recombinase‐aided amplification (rRT‐RAA) [92]	Detection of IAVs	China
RT‐RPA/CRISPR [93]	Detection of AIVs	China
Multiplex asymmetric RT‐PCR‐electrochemical DNA [94]	Simultaneous detection and subtyping of IAVs	China
xTAG‐multiplex PCR array [80]	Detection of avian influenza virus, Newcastle disease virus, infectious bronchitis virus, and infectious laryngotracheitis virus	China
Multiplex PCR and Matrix‐assisted laser desorption/ionization—time‐of‐flight mass spectrometry (MALDI‐TOF) [96]	Simultaneous detection and genotyping of 10 viruses in ducks including AIVs [96]	China

Insulated isothermal reverse transcriptase PCR methods were also developed for timely pathogen detection at sampling sites, such as insulated isothermal RT‐PCR for H7N9 [97, 98] with high sensitivity and specificity, and two insulated isothermal PCR devices, POCKIT DUO and POCKIT Central, and showed comparable performance to real‐time reverse transcription PCR for avian influenza detection [99].

Researchers developed reverse transcription loop‐mediated isothermal amplification (RT‐LAMP) diagnostic assays to detect a variety of zoonotic influenza viruses. A rapid and sensitive reverse RT‐LAMP assay was developed for detecting swine influenza virus in nasal samples, distinguishing between A(H1) and A(H3) strains directly from field samples without RNA extraction [100]. Three RT‐LAMP assays targeting the universal Matrix (M) gene, A(H5), and A(H9) HA for avian influenza showed higher sensitivity and faster amplification than conventional RT‐PCR [101]. One study described the application of RT‐LAMP to detect the M gene of IAVs in swine and showed very high sensitivity and specificity [102]. One study reported the rapid detection of seasonal and avian influenza viruses using RT‐LAMP technology with a one‐pot colorimetric visualization system, providing results within an hour [103]. A novel real‐time colorimetric RT‐LAMP assay was developed for the cost‐effective and rapid detection of avian influenza clade 2.3.4.4b H5 [104]. A highly efficient LAMP primer was developed for a real‐time RT‐LAMP assay that detects nine avian influenza subtypes and enables on‐site diagnosis without RNA extraction [105]. An integrated centrifugal RT‐LAMP disc was developed for the rapid detection of influenza A subtypes H1, H3, H5, H7, H9, and influenza B in clinical samples [106]. While nucleic amplification methods offer high sensitivity and specificity, they generally require trained personnel, controlled laboratory environments, and cold‐chain reagents, which may limit their application in low‐resource or field settings.

##### 3.2.4.2 Optical‐Based Methods

Surface‐enhanced Raman scattering–based assays have also shown applicability for the development of robust zoonotic influenza diagnostic tools [107, 108, 109]. Many immunochromatographic detection kits were developed for rapid and field detection of different subtypes of influenza viruses using novel monoclonal antibodies including H7N9 and H5Nx, highlighting their potential for effective surveillance and diagnosis in poultry and clinical settings [110, 111, 112, 113, 114, 115, 116, 117]. Few publications described fluorescent diagnostic assays that could detect simultaneously multiple influenza viruses, utilizing technologies like DNA‐templated silver nanoclusters [118], multiplex immunofluorescence platform based on ZnO nanorods [119], peptide‐linked systems to identify multiple virus subtypes efficiently [120], and label‐free imaging array capable of detecting simultaneously traces of three subtypes of AIVs DNA biomarkers (H1N1, H7N9, and H5N1) [121]. While optical‐based assays provide rapid and multiplexed detection, they often rely on specialized equipment, fluorescence labelling, or nanostructures, which can increase costs and reduce portability for field deployment.

##### 3.2.4.3 Nanoparticle‐Based Methods

Nanoparticle‐based methods detect influenza viruses by exploiting the unique optical, magnetic, or binding properties of nanoparticles, which change when they interact with viral proteins or nucleic acids [122]. These properties can then be measured using color change, fluorescence, or signal amplification, enabling rapid virus detection. Nanoparticle‐based methods were developed for the rapid detection of influenza viruses circulating in humans and animals that could be used in field surveillance [123, 124], including cell‐mimetic nanoparticles [125], cysteamine‐gold coated carboxylated europium chelated nanoparticle‐mediated dual‐mode point‐of‐care testing [126], glycan‐functionalized gold nanoparticles [127], combination peptide nucleic acid and unmodified gold nanoparticles [128], lateral flow immunoassays [129], and integrated magneto‐opto‐fluidic platform [130]. Nanoparticle‐based assays show promise for rapid and sensitive testing, yet they may require sophisticated instruments.

##### 3.2.4.4 Enzyme‐Linked Immunosorbent Assay–Based Methods

Various novel enzyme‐linked immunosorbent assay (ELISA) methods were developed from 2017 to 2024 for the rapid, sensitive, and specific detection of different avian influenza virus subtypes, utilizing monoclonal antibodies and innovative techniques to enhance diagnostic capabilities in laboratory and field settings [131, 132, 133, 134, 135, 136, 137, 138, 139, 140]. Those methods are summarized in Table 3. While traditional ELISA platforms are limited by cross‐reactivity, antigenic drift, and the need for monoclonal or polyclonal antibodies, the reviewed studies tried to overcome these obstacles by employing subtype‐specific antibodies, novel antigen‐capture formats, and innovative detection systems. Nevertheless, ELISA assays require ongoing optimization, particularly for field applications.

**TABLE 3:** ELISA methods developed from 2017 to 2024 for the detection of influenza viruses.

ELISA method	Type detected	Application
Sandwich ELISA [131]	HA of avian influenza A (H10N8)	Diagnostic of H10N8
AC‐ELISA and ICT [132]	H9N2 AIV	Field investigation of H9N2 subtype
DAS‐ELISA [133]	H9 viral antigen	H9 AIV diagnostic method
Sandwich ELISA [134]	H3 AIV	Detection of H3 AIV
Sandwich ELISA [134]	H4 AIV	Detection of H4 AIV
Sandwich ELISA [135]	NA of H7N9 AIV	Detection of H7N9 virus and quantification of N9 protein
ELASA [136]	Influenza A	Detection of Influenza A in analysis laboratories
AC‐ELISA [137]	HA of H6 AIV isolate	Diagnosis of H6 AIV infections
mAb sandwich ELISA [138]	Infectious diseases	Detection of infectious diseases
AC‐ELISA [139]	H7N9 AIV	Detection of H7N9
Ag‐ELISA [132]	H5 AIV	Detection of H5 AIV and field investigation of avian A(H5) viruses
Double‐antibody sandwich ELISA [133]	H7 AIV and quantification of H7 protein	Diagnosis and antigen quantification of H7 subtype
Antigen‐capture ELISA [140]	HA of H5 viruses	Rapid diagnosis of H5 virus infections

##### 3.2.4.5 Chip‐ and Probe‐Based Methods

Chip‐based methods were used to develop diagnostic kits with improved detection for use in biological warfare studies and for application on unpurified field samples [141, 142]. Probe‐based methods were developed to detect and amplify influenza viral sequences with high specificity and sensitivity even with degraded or low amount of viral RNA. Those methods include chemiluminescent lateral flow immunoassays that outperform commercial kits [143], universal influenza enrichment probes capable of detecting degraded viral RNA [144], as well as a fluorescent dye for rapid detection of avian influenza viruses by fluorescent immunochromatographic test [145]. A nucleic acid probe‐based electrochemical biosensor was developed to detect influenza A virus with enhanced sensitivity and selectivity [146]. Additionally, a hybridization capture probe panel was developed for effective avian influenza virus surveillance and subtyping [147]. A strand‐specific hybridization assay was developed capable of differentiating actual IAVs infection from environmental contamination [148]. A dual‐modality immunoassay to detect H9N2 AIV was developed based on integrating electrochemistry with fluorescence in one analytical system and showed high sensitivity and selectivity offering good potential in clinical diagnosis and disease treatment [149]. Chip‐ and probe‐based assays provide high specificity and multiplexing capacity but may require sophisticated fabrication processes, trained personnel, and specialized laboratory infrastructure.

##### 3.2.4.6 Microarray‐Based Methods

Microarray‐based methods were developed for the sensitive and specific detection of large number of targets in single assay, such as different subtypes of influenza viruses and the simultaneous detection of different viruses in animals. These methods utilize features like sequence‐specific molecular beacons [150], enhanced sensitivity through nanogold‐streptavidin and silver‐stain nucleic acid dot‐blot hybridization system [151], and glycan‐based microarray biosensors to differentiate between animal and human–infective strains [152]. The microarrays demonstrated significantly higher sensitivity compared to conventional PCR, allowing for rapid and accurate detection of multiple avian influenza subtypes and other respiratory viral diseases [153]. Additionally, new methods for detecting antibodies and studying antigenic properties of influenza viruses were developed, showcasing their potential for comprehensive viral surveillance [153, 154]. A multiplex label‐free arrayed imaging reflectometry platform was developed for studying antigenic properties of influenza viruses and showed ability to detect various subtypes of IAVs [155]. Microarray platforms enable simultaneous detection of multiple targets, but they remain relatively costly, technically complex, and are not easily adaptable to on‐site diagnostics.

##### 3.2.4.7 Electrical‐Based Methods

A few electrical‐based methods were developed and showed to be sensitive, fast, specific, and relatively cost‐effective. Peptide‐terminated electrodes were optimized to identify both seasonal and avian viruses [156], while a label‐free electrochemical paper‐based sensor demonstrated rapid detection of multiple AIVs antigens with good selectivity [157]. An enzyme‐free sandwich electrochemical immunosensor using graphene‐chitosan nanocomposites showed excellent performance for H9 AIVs detection providing a novel nonenzymatic method, which can be applied for the detection of other pathogens [158]. Additionally, a graphene oxide‐based immunosensor [159] and a graphene field effect transistor based ultrasensitive biosensor were developed [160], offering high specificity and sensitivity for avian influenza virus detection. These innovations highlight the potential for rapid, cost‐effective diagnostic solutions in clinical and field settings. Electrical biosensors offer speed and sensitivity, but many are still under development and face limitations in robustness, reproducibility, and validation for large‐scale surveillance use.

##### 3.2.4.8 Sequencing and Metagenomics Methods

Sequencing methods were enhanced for in‐field applications aiming at eliminating transportation challenges and direct on‐site sequencing and subtyping of influenza viruses from a variety of field samples and starting materials. Long read random sequencing, particularly using the Oxford Nanopore Technologies MinION platform [161], has proven effective for rapid identification and genetic characterization of respiratory pathogens in clinical samples [162]. A processing pipeline was developed to identify DNA and RNA viruses and bacteria from clinical samples within 12 h [163]. Studies show that amplicon‐based nanopore sequencing achieved complete genome coverage for all RT‐PCR–positive IAV samples tested offering complementary advantages to RT‐PCR such as genomic characterization and strain subtyping [164]. Additionally, a targeted sequence capture panel improved sensitivity and depth for detecting porcine viruses [165], and in‐field nanopore sequencing allowed timely characterization of avian influenza strains collected from wild bird feces and poultry [166]. One study reported the first successful application of metagenomic Nanopore sequencing directly to clinical respiratory samples to detect influenza virus [167]. Genomic surveillance of AIVs has emerged as a promising cost‐effective and robust approach to understand virus transmission and evolution and for informing outbreak control efforts and policies [168]. One study applied metagenomic technology to avian virus surveillance using short read sequencing with Ion Torrent and showed that this method could simultaneously detect major viruses infecting farms [169]. Sequencing and metagenomic approaches provide comprehensive genetic information, yet they usually require bioinformatics expertise.

##### 3.2.4.9 Commercial Panels and Developed Devices

A study showed that the QIAstat‐Dx Respiratory Panel could be used to detect zoonotic influenza A strains and differentiate them from the seasonal human seasonal IAVs [170]. Some research groups focused on developing devices for the detection of various diseases in a sensitive, specific, and timely manner, and with minimum sample handling and laboratory skill requirements.

One study validated a novel point of care diagnostic device utilizing photonic integrated circuits (PICs), microfluidics, and information and communication technologies for the detection of swine IAVs using spiked and clinical oral fluid samples and reported high sensitivity and specificity [171]. Another portable quantitative device based on giant magnetoresistive (GMR) technology was developed for detecting sensitively and specifically swine IAVs with minimum sample handling and laboratory skill requirements [172]. Commercial panels and portable devices can simplify diagnostics, but the need for validation across diverse field conditions may restrict widespread adoption.

#### 3.2.5 Effectiveness in Improving Risk Assessment on Human Infection by Animal IAVs

Readily available the WOAH‐World Animal Health Information System (WAHIS), public surveillance data and time‐series models were used to build an early warning model to predict the detection of H5 HPAI with two seasonal waves in the endemic component and three seasonal waves in the epidemic component. The early warning model showed flexibility to be used even in countries with large temporal variation in the number of HPAI detections to predict detections. This model is capable of monitoring the weekly risk of HPAI outbreaks within European countries to support decision making and timely implementation of preventive measures [173].

## 4 Discussion

The 2017 WHO public health research agenda for influenza recommended the development of more sensitive and specific influenza virus surveillance and detection systems in farmed animals and wildlife, including epidemiological designs and novel technologies. Since 2017, many countries have developed novel influenza surveillance systems or have introduced enhancements to their existing surveillance programs to improve monitoring of influenza viruses and enhance early detection and response. In this report, we summarized enhanced and novel surveillance methods and technologies applied in domestic animal husbandry and exposed humans and in wildlife and their outcomes and highlighted gaps. While this review focused specifically on novel and enhanced surveillance methodologies, these findings also indirectly reflect the broader status of zoonotic influenza surveillance by highlighting how methodological advances have addressed some gaps while leaving others unresolved.

In domestic animals, over the last 8 years, few predictive surveillance systems were developed focusing mainly on assessing the risk of introduction of zoonotic influenza into domestic animals from contact with wild animals using influenza transmission, demographic, and spatial risk factors and few research studies reported participatory community‐based surveillance systems for emerging zoonoses incorporating demographic and behavioral factors under the One Health approach. Surveillance systems in domestic animals have gained scientists' interest during the last decade and enhancements have been introduced, especially focusing on environmental noninvasive sampling approaches. Integrating data management, bioinformatics, and biosafety practices should be considered by the research community for the development of effective surveillance systems as well as combining surveillance data within risk assessment frameworks to better assess zoonotic and pandemic potential of novel IAVs strains and to inform mitigation measures. Research studies conducted to develop One Health surveillance systems were sparse. Thus, more attention should be given to One Health surveillance systems by integrating human, animal, and environmental data. This need for integrated, forward‐looking surveillance systems aligns with the priorities highlighted in recent WHO R&D Blueprint consultations on H5N1, which emphasized the urgency of strengthening genomic surveillance, rapid data sharing, and the development of candidate vaccines, therapeutics, and diagnostics to prepare for potential pandemic spread [174].

Only two digital dashboards have been developed as analytical interactive tools for zoonotic influenza monitoring aiming at enhancing the accessibility of data and information for policy makers and stakeholders to formulate policy and ensure appropriate mitigation measures. Research should focus on the use of mobile health technologies and smart applications for reporting of infections in humans and engage communities in surveillance efforts.

In wild animals, few predictive surveillance systems were developed for monitoring and isolating zoonotic influenza from wild birds by studying geographic factors, spatiotemporal models, ecological nature of wild birds, and observing the health of wild birds. A few sampling approaches in wild birds were developed that aimed at improving early detection of zoonotic influenza in wild birds to protect local animal population focusing on minimizing invasive methods. Novel environmental sampling methods were developed globally from 2017 to 2024 to improve the detection of influenza viruses in wild animals, improve monitoring of those viruses, and creating transmission risk maps. Research should focus on making those novel environmental surveillance methods useful to public health authorities. Surveillance should be increased in animal populations through using risk assessment studies and findings to implement regular sampling and surveillance of wild birds and domestic animals in high‐risk areas. Furthermore, the utilization of risk assessment tools and mathematical modelling should be enhanced to anticipate the spread of zoonotic IAVs and predict the emergence of novel influenza strains using environmental, demographic, and epidemiological data.

Innovations in field and laboratory testing for influenza viruses have significantly bolstered both animal and human surveillance. Advances in molecular techniques, particularly RT‐qPCR and next‐generation sequencing, have improved the rapid detection and genetic analysis of viral strains. Additionally, the emergence of next‐generation sequencing and genomic surveillance has facilitated the genetic analysis of viral strains, aiding in understanding their origins and spill‐over risks. Sensitive nucleic acid amplification methods, multiplex PCR, and insulated isothermal RT‐PCR enhance diagnostic capabilities. Further developments include RT‐LAMP assays, surface‐enhanced Raman spectroscopy–based methods, and immunochromatographic kits for rapid detection. ELISA methods using monoclonal antibodies and chip‐based diagnostics were developed to improve detection for diverse applications. Enhanced sequencing methods facilitate in‐field applications, enabling direct on‐site analysis of influenza viruses. Additionally, an early warning model utilizing public surveillance data has been developed to predict HPAI outbreaks in Europe, supporting timely preventive measures. Overall, these advancements enhance the capacity for effective influenza surveillance and response. Although RT‐PCR remains the gold standard technique for sensitivity and specificity, the new technologies described in this review offer complementary advantages such as faster turnaround times, reduced infrastructure requirements, multiplexing capacity or true portability in the field. Pursuing these technologies is therefore important to strengthen decentralized surveillance, improve outbreak response in resource‐limited settings, and provide additional layers of data to guide risk assessment. Updating developed molecular‐based diagnosis tests for zoonotic influenza regularly to cover the continuous genetic assortment of zoonotic, swine, and avian influenza viruses and combining molecular‐based techniques with nanotechnology to develop new diagnostic tests for zoonotic influenza are recommended. Sequencing methods were enhanced for in‐field applications aiming at eliminating transportation challenges and direct on‐site sequencing and subtyping of influenza viruses from a variety of field samples and starting materials. These methods have also been applied for multiplexing and for metagenomics for the detection of DNA and RNA viruses and bacterial pathogens from clinical samples. Additional research should be focused on enhanced genomic surveillance and the development of portable sequencing technologies for on‐site rapid identification of viral strains in remote areas and wildlife. Moreover, big data analytics and machine learning algorithms should be applied in addition to digital health records to monitor trends in influenza occurrence and detect anomalies and predict outbreaks. Some research groups focused on developing devices for the detection of various diseases in a sensitive, specific, and timely manner, and with minimum sample handling and laboratory skill requirements. Such devises could be used as fieldable, cost‐effective tools to complement laboratory‐based gold standard assays, especially where rapid decision making is needed. Research should focus on developing new rapid, sensitive, and inexpensive portable detection kits for field influenza virus detection and surveillance with subtyping capacities. Taken together, the methodologies reviewed vary in their suitability for different surveillance contexts. Nucleic acid amplification assays and RT‐qPCR remain best suited for laboratory‐based diagnosis, while isothermal methods (e.g., RT‐LAMP, insulated isothermal PCR) hold promise for use in resource‐limited or field settings. Optical‐ and nanoparticle‐based technologies demonstrate high sensitivity in proof‐of‐concept studies but require further validation before widespread adoption. Sequencing approaches, while generally less sensitive for low viral loads, provide essential genomic data that is particularly valuable for risk assessment and outbreak investigations. For wildlife surveillance, noninvasive sampling approaches are most practical and ethically feasible, whereas for farm settings, pooled environmental and group sampling methods have shown the greatest efficiency.

One early warning model using available WOAH‐WAHIS HPAI public surveillance data and time‐series models was developed to predict the detection of A(H5) HPAI and monitor the weekly risk of HPAI outbreaks within European countries to support decision making and timely implementation of preventive measures. More research on assessing the effectiveness of improving risk assessment on human infection by animal influenza A viruses should be conducted. Enhancing environmental monitoring of influenza viruses and applying remote sensing technologies to track migratory patterns of influenza virus reservoir are recommended.

The key gaps highlighted by this review include insufficient integration of One Health data streams, limited application of noninvasive and environmental sampling approaches, inadequate validation and deployment of emerging diagnostic technologies, and sparse evaluation of how novel methodologies inform risk assessment and decision making. Addressing these gaps should be prioritized in future research and international surveillance initiatives.

A limitation of this review is the reliance on PubMed as the primary database, which may have biased the included literature toward English‐language publications and internationally indexed journals. As a result, surveillance studies reported in local or regional journals, particularly in non‐English languages, may have been missed. This limitation could partially explain the underrepresentation of certain geographic regions in the included studies.

## 5 Conclusions

The evolution of influenza surveillance systems since the 2017 WHO public health research agenda update has led to advancements in monitoring both domestic animals and wildlife. Analysis of the collected publications indicated several gaps at different levels of surveillance and unmet public health needs. Most countries do not have active and comprehensive surveillance programs for zoonotic influenza at the human–animal interface, which underestimates the true burden of zoonotic influenza diseases. This conclusion is supported by the geographic distribution of studies included in this review, which shows concentrations in East/Southeast Asia, Europe, and North America, but very limited representation from Africa, South America, and the Middle East. Although there has been progress in developing predictive surveillance systems and integrating innovative technologies, notable gaps remain, particularly in the context of One Health approaches that encompass human, animal, and environmental data. The development of participatory community‐based surveillance and the use of digital tools, such as mobile health technologies, are essential for engaging communities and improving data accessibility for policymakers.

Further research is needed to refine and enhance the effectiveness of these surveillance systems, particularly through risk assessment frameworks that can anticipate zoonotic threats. The novel environmental sampling methods and advances in molecular diagnostics, such as real‐time PCR and next‐generation sequencing, have bolstered detection capabilities but require ongoing updates to account for the genetic diversity of influenza viruses. Moreover, the application of big data analytics and machine learning could improve the predictive power of surveillance systems, allowing for timely interventions. Emphasizing the importance of portable and cost‐effective diagnostic tools will facilitate rapid field detection, enhancing our capacity to respond to outbreaks effectively. Our findings underscore the need to focus on filling gaps in One Health integration, validation and field deployment of novel assays, expansion of noninvasive and environmental surveillance, and stronger linkage of methodological innovations to risk assessment and policy action. As we move forward, prioritizing comprehensive surveillance efforts will be crucial in mitigating the risks posed by zoonotic influenza and safeguarding public health under the One Health approach.

## Author Contributions

**Rebecca Badra:** conceptualization, methodology, validation, data curation, writing – original draft, writing – review and editing. **Wenqing Zhang:** conceptualization, funding acquisition, project administration, writing – review and editing. **John S. L. Tam:** conceptualization, writing – review and editing. **Richard Webby:** writing – review and editing. **Sylvie Van Der Werf:** writing – review and editing. **Sergejs Nikisins:** project administration, writing – review and editing, resources. **Ann Cullinane:** writing – review and editing. **Saad Gharaibeh:** writing – review and editing. **Richard Njouom:** writing – review and editing. **Malik Peiris:** writing – review and editing. **Ghazi Kayali:** conceptualization, methodology, data curation, writing – review and editing, supervision. **Jean‐Michel Heraud:** conceptualization, supervision, project administration, writing – review and editing.

## Conflicts of Interest

The authors declare no conflicts of interest.

## Data Availability

All data are present in the manuscript.

## References

[1] S. Kessler , T. C. Harder , M. Schwemmle , and K. Ciminski , “Influenza A Viruses and Zoonotic Events‐Are We Creating Our Own Reservoirs?,” Viruses 13, no. 11 (2021): 2250. 34835056 10.3390/v13112250 PMC8624301

[2] S. Al Hajjar and K. McIntosh , “The First Influenza Pandemic of the 21st Century,” Annals of Saudi Medicine 30, no. 1 (2010): 1–10. 20103951 10.4103/0256-4947.59365 PMC2850175

[3] M. Lemaitre and F. Carrat , “Comparative Age Distribution of Influenza Morbidity and Mortality During Seasonal Influenza Epidemics and the 2009 H1N1 Pandemic,” BMC Infectious Diseases 10 (2010): 162. 20534113 10.1186/1471-2334-10-162 PMC2896934

[4] A. Findlater and I. I. Bogoch , “Human Mobility and the Global Spread of Infectious Diseases: A Focus on Air Travel,” Trends in Parasitology 34, no. 9 (2018): 772–783. 30049602 10.1016/j.pt.2018.07.004 PMC7106444

[5] M. E. Manuell , M. D. Co , and R. T. Ellison, III , “Pandemic Influenza: Implications for Preparation and Delivery of Critical Care Services,” Journal of Intensive Care Medicine 26, no. 6 (2011): 347–367. 21220275 10.1177/0885066610393314 PMC4110744

[6] C.‐Y. Lee , “Exploring Potential Intermediates in the Cross‐Species Transmission of Influenza A Virus to Humans,” Viruses 16, no. 7 (2024): 1129. 39066291 10.3390/v16071129 PMC11281536

[7] M. Wille and E. C. Holmes , “The Ecology and Evolution of Influenza Viruses,” Cold Spring Harbor Perspectives in Medicine 10, no. 7 (2020): a038489. 31871237 10.1101/cshperspect.a038489 PMC7328453

[8] H. Liao , C. J. Lyon , B. Ying , and T. Hu , “Climate Change, Its Impact on Emerging Infectious Diseases and New Technologies to Combat the Challenge,” Emerging Microbes & Infections 13, no. 1 (2024): 2356143. 38767202 10.1080/22221751.2024.2356143 PMC11138229

[9] C. C. Sreenivasan , F. Li , and D. Wang , “Emerging Threats of Highly Pathogenic Avian Influenza A (H5N1) in US Dairy Cattle: Understanding Cross‐Species Transmission Dynamics in Mammalian Hosts,” Viruses 16, no. 11 (2024): 1703. 39599818 10.3390/v16111703 PMC11598956

[10] E. R. Burrough , D. R. Magstadt , B. Petersen , et al., “Highly Pathogenic Avian Influenza A(H5N1) Clade 2.3.4.4b Virus Infection in Domestic Dairy Cattle and Cats, United States, 2024,” Emerging Infectious Diseases 30, no. 7 (2024): 1335–1343. 38683888 10.3201/eid3007.240508 PMC11210653

[11] T. M. Uyeki , S. Milton , C. A. Hamid , et al., “Highly Pathogenic Avian Influenza A(H5N1) Virus Infection in a Dairy Farm Worker,” New England Journal of Medicine 390, no. 21 (2024): 2028–2029. 38700506 10.1056/NEJMc2405371

[12] World Health Organization . Preparing for Containment and Mitigation of Pandemic H5N1 Influenza: Uses of Statistical and Mathematical Modeling (2024), https://www.who.int/news‐room/events/detail/2024/11/14/default‐calendar/preparing‐for‐containment‐and‐mitigation‐of‐pandemic‐h5n1‐influenza‐‐uses‐of‐statistical‐and‐mathematical‐modeling.

[13] E. K. Lee , Y. Liu , and F. H. Pietz , “A Computational Framework for a Digital Surveillance and Response Tool: Application to Avian Influenza,” American Medical Informatics Association Annual Symposium Proceedings 2017 (2017): 1090–1099. PMC5977687 29854177

[14] G. Galletti , A. Santi , V. Guberti , et al., “A Method to Identify the Areas at Risk for the Introduction of Avian Influenza Virus Into Poultry Flocks Through Direct Contact With Wild Ducks,” Transboundary and Emerging Diseases 65, no. 4 (2018): 1033–1038. 29473322 10.1111/tbed.12838

[15] A. Hill , S. Gillings , A. Berriman , et al., “Quantifying the Spatial Risk of Avian Influenza Introduction Into British Poultry by Wild Birds,” Scientific Reports 9, no. 1 (2019): 19973. 31882592 10.1038/s41598-019-56165-9 PMC6934731

[16] J. Schreuder , H. J. de Knegt , F. C. Velkers , et al., “Wild Bird Densities and Landscape Variables Predict Spatial Patterns in HPAI Outbreak Risk Across The Netherlands,” Pathogens 11, no. 5 (2022): 549. 35631070 10.3390/pathogens11050549 PMC9143584

[17] T. Petukhova , D. Ojkic , B. McEwen , R. Deardon , and Z. Poljak , “Assessment of Autoregressive Integrated Moving Average (ARIMA), Generalized Linear Autoregressive Moving Average (GLARMA), and Random Forest (RF) Time Series Regression Models for Predicting Influenza A Virus Frequency in Swine in Ontario, Canada,” PLoS ONE 13, no. 6 (2018): e0198313. 29856881 10.1371/journal.pone.0198313 PMC5983852

[18] D. S. Yoo , Y. H. Song , D. W. Choi , J. S. Lim , K. Lee , and T. Kang , “Machine Learning‐Driven Dynamic Risk Prediction for Highly Pathogenic Avian Influenza at Poultry Farms in Republic of Korea: Daily Risk Estimation for Individual Premises,” Transboundary and Emerging Diseases 69, no. 5 (2022): 2667–2681. 34902223 10.1111/tbed.14419

[19] S. Yousefinaghani , R. Dara , Z. Poljak , F. Song , and S. Sharif , “A Framework for the Risk Prediction of Avian Influenza Occurrence: An Indonesian Case Study,” PLoS ONE 16, no. 1 (2021): e0245116. 33449934 10.1371/journal.pone.0245116 PMC7810353

[20] S. M. Thumbi , M. K. Njenga , E. Otiang , et al., “Mobile Phone‐Based Surveillance for Animal Disease in Rural Communities: Implications for Detection of Zoonoses Spillover,” Philosophical Transactions of the Royal Society of London. Series B, Biological Sciences 374, no. 1782 (2019): 20190020. 31401960 10.1098/rstb.2019.0020 PMC6711315

[21] T. Yano , S. Phornwisetsirikun , P. Susumpow , et al., “A Participatory System for Preventing Pandemics of Animal Origins: Pilot Study of the Participatory One Health Disease Detection (PODD) System,” JMIR Public Health and Surveillance 4, no. 1 (2018): e25. 29563079 10.2196/publichealth.7375 PMC5885059

[22] S. Yadana , T. Cheun‐Arom , H. Li , et al., “Behavioral‐Biological Surveillance of Emerging Infectious Diseases Among a Dynamic Cohort in Thailand,” BMC Infectious Diseases 22, no. 1 (2022): 472. 35578171 10.1186/s12879-022-07439-7 PMC9109443

[23] S. Munaf , K. Swingler , F. Brulisauer , A. O'Hare , G. Gunn , and A. Reeves , “Spatio‐Temporal Evaluation of Social Media as a Tool for Livestock Disease Surveillance,” One Health 17 (2023): 100657. 38116453 10.1016/j.onehlt.2023.100657 PMC10728316

[24] J. Garrido‐Mantilla , J. Alvarez , M. Culhane , J. Nirmala , J. P. Cano , and M. Torremorell , “Comparison of Individual, Group and Environmental Sampling Strategies to Conduct Influenza Surveillance in Pigs,” BMC Veterinary Research 15, no. 1 (2019): 61. 30764815 10.1186/s12917-019-1805-0 PMC6376652

[25] A. C. de Lara , J. Garrido‐Mantilla , G. Lopez‐Moreno , M. Yang , D. Barcellos , and M. Torremorell , “Effect of Pooling Udder Skin Wipes on the Detection of Influenza A Virus in Preweaning Pigs,” Journal of Veterinary Diagnostic Investigation 34, no. 1 (2022): 133–135. 34404296 10.1177/10406387211039462 PMC8689042

[26] K. Prost , H. Kloeze , S. Mukhi , K. Bozek , Z. Poljak , and S. Mubareka , “Bioaerosol and Surface Sampling for the Surveillance of Influenza A Virus in Swine,” Transboundary and Emerging Diseases 66, no. 3 (2019): 1210–1217. 30715792 10.1111/tbed.13139

[27] B. D. Anderson , M. Yondon , E. S. Bailey , et al., “Environmental Bioaerosol Surveillance as an Early Warning System for Pathogen Detection in North Carolina Swine Farms: A Pilot Study,” Transboundary and Emerging Diseases 68, no. 2 (2021): 361–367. 32535997 10.1111/tbed.13683

[28] P. Bessiere , B. Hayes , F. Filaire , et al., “Optimizing Environmental Viral Surveillance: Bovine Serum Albumin Increases RT‐qPCR Sensitivity for High Pathogenicity Avian Influenza H5Nx Virus Detection From Dust Samples,” Microbiology Spectrum 11, no. 6 (2023): e0305523. 37982626 10.1128/spectrum.03055-23 PMC10715206

[29] D. Wade , A. Ashton‐Butt , G. Scott , et al., “High Pathogenicity Avian Influenza: Targeted Active Surveillance of Wild Birds to Enable Early Detection of Emerging Disease Threats,” Epidemiology and Infection 151 (2022): e15. 36502812 10.1017/S0950268822001856 PMC9990394

[30] C. Abolnik , T. P. Phiri , G. van der Zel , J. Anthony , N. Daniell , and L. de Boni , “Wild Bird Surveillance in the Gauteng Province of South Africa During the High‐Risk Period for Highly Pathogenic Avian Influenza Virus Introduction,” Viruses 14, no. 9 (2022): 2027. 36146838 10.3390/v14092027 PMC9504564

[31] J. V. Camp , A. Desvars‐Larrive , N. Nowotny , and C. Walzer , “Monitoring Urban Zoonotic Virus Activity: Are City Rats a Promising Surveillance Tool for Emerging Viruses?,” Viruses 14, no. 7 (2022): 1516. 35891496 10.3390/v14071516 PMC9316102

[32] A. Ssematimba , P. J. Bonney , S. Malladi , et al., “Mortality‐Based Triggers and Premovement Testing Protocols for Detection of Highly Pathogenic Avian Influenza Virus Infection in Commercial Upland Game Birds,” Avian Diseases 63, no. sp1 (2019): 157–164. 31131573 10.1637/11870-042518-Reg.1

[33] N. S. Trovao , J. M. Nolting , R. D. Slemons , M. I. Nelson , and A. S. Bowman , “The Evolutionary Dynamics of Influenza A Viruses Circulating in Mallards in Duck Hunting Preserves in Maryland, USA,” Microorganisms 9, no. 1 (2020): 40. 33375548 10.3390/microorganisms9010040 PMC7823399

[34] F. Capelastegui , J. Smith , J. Kumbang , et al., “Pilot of Asymptomatic Swabbing of Humans Following Exposures to Confirmed Avian Influenza A(H5) in Avian Species in England, 2021/2022,” Influenza and Other Respiratory Viruses 17, no. 8 (2023): e13187. 37638093 10.1111/irv.13187 PMC10447230

[35] L. C. Tindale , W. Baticados , J. Duan , et al., “Extraction and Detection of Avian Influenza Virus From Wetland Sediment Using Enrichment‐Based Targeted Resequencing,” Frontiers in Veterinary Science 7 (2020): 301. 32548133 10.3389/fvets.2020.00301 PMC7273442

[36] W. Yamazaki , R. Makino , K. Nagao , H. Mekata , and K. Tsukamoto , “New Micro‐Amount of Virion Enrichment Technique (MiVET) to Detect Influenza A Virus in the Duck Faeces,” Transboundary and Emerging Diseases 66, no. 1 (2019): 341–348. 30267611 10.1111/tbed.13027

[37] G. K. Innes , A. S. Lambrou , P. Thumrin , et al., “Enhancing Global Health Security in Thailand: Strengths and challenges of Initiating a One Health Approach to Avian Influenza Surveillance,” One Health 14 (2022): 100397. 35686140 10.1016/j.onehlt.2022.100397 PMC9171517

[38] M. A. Zeller , T. K. Anderson , R. W. Walia , A. L. Vincent , and P. C. Gauger , “ISU FLUture: A Veterinary Diagnostic Laboratory Web‐Based Platform to Monitor the Temporal Genetic Patterns of Influenza A Virus in Swine,” BMC Bioinformatics 19, no. 1 (2018): 397. 30382842 10.1186/s12859-018-2408-7 PMC6211438

[39] Z. W. Arendsee , J. Chang , D. E. Hufnagel , et al., “OctoFLUshow: An Interactive Tool Describing Spatial and Temporal Trends in the Genetic Diversity of Influenza A Virus in U.S. Swine,” Microbiology Resource Announcements 10, no. 50 (2021): e0108121. 34913720 10.1128/MRA.01081-21 PMC8675265

[40] D. P. Walsh , T. F. Ma , H. S. Ip , and J. Zhu , “Artificial Intelligence and Avian Influenza: Using Machine Learning to Enhance Active Surveillance for Avian Influenza Viruses,” Transboundary and Emerging Diseases 66, no. 6 (2019): 2537–2545. 31376332 10.1111/tbed.13318

[41] I. Saavedra , J. Rabadan‐Gonzalez , D. Aragones , and J. Figuerola , “Can Citizen Science Contribute to Avian Influenza Surveillance?,” Pathogens 12, no. 9 (2023): 1183. 37764991 10.3390/pathogens12091183 PMC10535995

[42] H. I. Wu , R. S. Lin , W. H. Hwang , et al., “Integrating Citizen Scientist Data Into the Surveillance System for Avian Influenza Virus, Taiwan,” Emerging Infectious Diseases 29, no. 1 (2023): 45–53. 36573518 10.3201/eid2901.220659 PMC9796195

[43] D. S. Yoo , K. Lee , M. L. Beatriz , B. C. Chun , J. Belkhiria , and K. N. Lee , “Spatiotemporal Risk Assessment for Avian Influenza Outbreak Based on the Dynamics of Habitat Suitability for Wild Birds,” Transboundary and Emerging Diseases 69, no. 4 (2022): e953–e967. 34738338 10.1111/tbed.14376

[44] K. Nabeshima , S. Asakura , R. Iwata , et al., “Sequencing Methods for HA and NA Genes of Avian Influenza Viruses From Wild Bird Feces Using Oxford Nanopore Sequencing,” Comparative Immunology, Microbiology and Infectious Diseases 102 (2023): 102076. 37804607 10.1016/j.cimid.2023.102076

[45] H. Pereira , M. Artois , and D. J. Bicout , “Fireworks‐Like Surveillance Approach: The Case of HPAI H5N1 in Wild Birds in Europe,” Transboundary and Emerging Diseases 67, no. 1 (2020): 206–222. 31482660 10.1111/tbed.13342

[46] M. M. McCuen , M. E. Pitesky , J. J. Buler , et al., “A Comparison of Amplification Methods to Detect Avian Influenza Viruses in California Wetlands Targeted via Remote Sensing of Waterfowl,” Transboundary and Emerging Diseases 68, no. 1 (2021): 98–109. 32592444 10.1111/tbed.13612 PMC8048853

[47] M. Vittecoq , H. Gauduin , T. Oudart , et al., “Modeling the Spread of Avian Influenza Viruses in Aquatic Reservoirs: A Novel Hydrodynamic Approach Applied to the Rhone Delta (Southern France),” Science of the Total Environment 595 (2017): 787–800. 28410528 10.1016/j.scitotenv.2017.03.165

[48] E. A. Germeraad , A. R. W. Elbers , N. D. de Bruijn , et al., “Detection of Low Pathogenic Avian Influenza Virus Subtype H10N7 in Poultry and Environmental Water Samples During a Clinical Outbreak in Commercial Free‐Range Layers, Netherlands 2017,” Frontiers in Veterinary Science 7 (2020): 237. 32478107 10.3389/fvets.2020.00237 PMC7232570

[49] S. Azeem , B. Guo , Y. Sato , P. C. Gauger , A. Wolc , and K. J. Yoon , “Utility of Feathers for Avian Influenza Virus Detection in Commercial Poultry,” Pathogens 12, no. 12 (2023): 1425. 38133308 10.3390/pathogens12121425 PMC10748246

[50] L. F. La Sala , J. M. Burgos , D. E. Blanco , et al., “Spatial Modelling for Low Pathogenicity Avian Influenza Virus at the Interface of Wild Birds and Backyard Poultry,” Transboundary and Emerging Diseases 66, no. 4 (2019): 1493–1505. 30698918 10.1111/tbed.13136

[51] K. A. Berger , D. M. Pigott , F. Tomlinson , et al., “The Geographic Variation of Surveillance and Zoonotic Spillover Potential of Influenza Viruses in Domestic Poultry and Swine. Open Forum,” Infectious Diseases 5, no. 12 (2018): ofy318. 10.1093/ofid/ofy318 PMC6309522 30619908

[52] G. Hood , X. Roche , A. Brioudes , et al., “A Literature Review of the Use of Environmental Sampling in the Surveillance of Avian Influenza Viruses,” Transboundary and Emerging Diseases 68, no. 1 (2021): 110–126. 32652790 10.1111/tbed.13633 PMC8048529

[53] M. H. Lee , K. Y. Song , H. J. Hwang , J. H. Kim , and I. Hwang , “Development of Fast and Sensitive Protocols for the Detection of Viral Pathogens Using a Small Portable Convection PCR Platform,” Molecular Biology Reports 46, no. 5 (2019): 5073–5077. 31313130 10.1007/s11033-019-04961-x

[54] Z. Wang , P. P. Yang , Y. H. Zhang , K. Y. Tian , C. Z. Bian , and J. Zhao , “Development of a Reverse Transcription Recombinase Polymerase Amplification Combined With Lateral‐Flow Dipstick Assay for Avian Influenza H9N2 HA Gene Detection,” Transboundary and Emerging Diseases 66, no. 1 (2019): 546–551. 30403438 10.1111/tbed.13063

[55] S. Ma , X. Li , B. Peng , et al., “Rapid Detection of Avian Influenza A Virus (H7N9) by Lateral Flow Dipstick Recombinase Polymerase Amplification,” Biological & Pharmaceutical Bulletin 41, no. 12 (2018): 1804–1808. 30232304 10.1248/bpb.b18-00468

[56] Z. Zhang , Z. Zhang , C. Wang , et al., “Detection Method for Reverse Transcription Recombinase‐Aided Amplification of Avian Influenza Virus Subtypes H5, H7, and H9,” BMC Veterinary Research 20, no. 1 (2024): 203. 38755641 10.1186/s12917-024-04040-9 PMC11097555

[57] F. Zhang , J. Shang , J. Luo , et al., “Development of a Recombinase‐Aided Amplification Combined With a Lateral Flow Dipstick Assay for Rapid Detection of H7 Subtype Avian Influenza Virus,” Frontiers in Microbiology 14 (2023): 1286713. 38029110 10.3389/fmicb.2023.1286713 PMC10654746

[58] Y. Wu , J. Zhan , Z. Shan , et al., “CRISPR‐Cas13a‐Based Detection Method for Avian Influenza Virus,” Frontiers in Microbiology 14 (2023): 1288951. 37886067 10.3389/fmicb.2023.1288951 PMC10598603

[59] F. Yang , S. Yan , L. Zhu , et al., “A Multiplex TaqMan Real‐Time RT‐PCR Assay for the Simultaneous Detection of H4, H6, and H10 Avian Influenza Viruses,” Heliyon 9, no. 5 (2023): e15647. 37153423 10.1016/j.heliyon.2023.e15647 PMC10160747

[60] E. Spackman , “Avian Influenza Virus Detection and Quantitation by Real‐Time RT‐PCR,” in Methods in Molecular Biology, vol. 2123, (Springer, 2020), 137–148. 32170686 10.1007/978-1-0716-0346-8_11

[61] S. G. Mirzaei , A. Shoushtari , and A. Nouri , “Development and Evaluation of Real‐Time RT‐PCR Test for Quantitative and Qualitative Recognition of Current H9N2 Subtype Avian Influenza Viruses in Iran,” Archives of Razi Institute 73, no. 3 (2018): 177–182. 30280837 10.22092/ari.2017.106968.1059

[62] Y. Xiao , F. Yang , F. Liu , H. Yao , N. Wu , and H. Wu , “Development and Application of a Real‐Time RT‐PCR Assay to Rapidly Detect H2 Subtype Avian Influenza A Viruses,” Journal of Veterinary Diagnostic Investigation 33, no. 3 (2021): 577–581. 33618630 10.1177/1040638721994810 PMC8120088

[63] M. Sagong , Y. M. Kang , N. Y. Kim , et al., “Development of a Novel Korean H9‐Specific rRT‐PCR Assay and Its Application for Avian Influenza Virus Surveillance in Korea,” Journal of Microbiology 61, no. 10 (2023): 929–936. 38010587 10.1007/s12275-023-00088-8

[64] S. G. Mirzaei , A. Shoushtari , and A. Nouri , “Development and Evaluation of Real‐Time Reverse Transcription Polymerase Chain Reaction Test for Quantitative and Qualitative Recognition of H5 Subtype of Avian Influenza Viruses,” Archives of Razi Institute 75, no. 1 (2020): 17–22. 32291998 10.22092/ari.2019.120821.1201 PMC8410168

[65] J. James , A. H. Seekings , P. Skinner , et al., “Rapid and Sensitive Detection of High Pathogenicity Eurasian Clade 2.3.4.4b Avian Influenza Viruses in Wild Birds and Poultry,” Journal of Virological Methods 301 (2022): 114454. 34998830 10.1016/j.jviromet.2022.114454

[66] F. Yang , D. Dong , D. Wu , et al., “A Multiplex Real‐Time RT‐PCR Method for Detecting H5, H7 and H9 Subtype Avian Influenza Viruses in Field and Clinical Samples,” Virus Research 309 (2022): 198669. 34954007 10.1016/j.virusres.2021.198669

[67] D. Henritzi , B. Hoffmann , S. Wacheck , et al., “A Newly Developed Tetraplex Real‐Time RT‐PCR for Simultaneous Screening of Influenza Virus Types A, B, C and D,” Influenza and Other Respiratory Viruses 13, no. 1 (2019): 71–82. 30264926 10.1111/irv.12613 PMC6304318

[68] X. Zhang , M. Yao , Z. Tang , et al., “Development and Application of a Triplex Real‐Time PCR Assay for Simultaneous Detection of Avian Influenza Virus, Newcastle Disease Virus, and Duck Tembusu Virus,” BMC Veterinary Research 16, no. 1 (2020): 203. 32560692 10.1186/s12917-020-02399-z PMC7304117

[69] F. Wen , C. Wang , J. Guo , et al., “Development and Application of a Triplex Real‐Time PCR Assay for the Detection of H3, H4, and H5 Subtypes of Avian Influenza Virus,” Poultry Science 103, no. 2 (2024): 103333. 10.1016/j.psj.2023.103333 PMC10770746 38113705

[70] J. Liu , L. Yao , F. Zhai , et al., “Development and Application of a Triplex Real‐Time PCR Assay for the Simultaneous Detection of Avian Influenza Virus Subtype H5, H7 and H9,” Journal of Virological Methods 252 (2018): 49–56. 29129489 10.1016/j.jviromet.2017.11.005

[71] F. Yang , B. Chen , F. Liu , et al., “Development of a TaqMan MGB RT‐PCR Assay for the Detection of Type A and Subtype H10 Avian Influenza Viruses,” Archives of Virology 163, no. 9 (2018): 2497–2501. 29796926 10.1007/s00705-018-3889-4

[72] T. B. Le , H. K. Kim , W. Na , et al., “Development of a Multiplex RT‐qPCR for the Detection of Different Clades of Avian Influenza in Poultry,” Viruses 12, no. 1 (2020): 100. 31952218 10.3390/v12010100 PMC7019278

[73] Y. Yang , S. Li , G. Wong , et al., “Development of a Quadruple qRT‐PCR Assay for Simultaneous Identification of Highly and Low Pathogenic H7N9 Avian Influenza Viruses and Characterization Against Oseltamivir Resistance,” BMC Infectious Diseases 18, no. 1 (2018): 406. 30111290 10.1186/s12879-018-3302-7 PMC6094886

[74] L. Liu , Y. Zhang , P. Cui , et al., “Development of a Duplex TaqMan Real‐Time RT‐PCR Assay for Simultaneous Detection of Newly Emerged H5N6 Influenza Viruses,” Virology Journal 16, no. 1 (2019): 119. 31640801 10.1186/s12985-019-1229-2 PMC6805314

[75] Z. Sun , T. Qin , F. Meng , S. Chen , D. Peng , and X. Liu , “Development of a Multiplex Probe Combination‐Based One‐Step Real‐Time Reverse Transcription‐PCR for NA Subtype Typing of Avian Influenza Virus,” Scientific Reports 7, no. 1 (2017): 13455. 29044197 10.1038/s41598-017-13768-4 PMC5647442

[76] F. Yang , H. Wu , F. Liu , X. Lu , X. Peng , and N. Wu , “Establishment of a Multiplex Real‐Time RT‐PCR Assay for Rapid Identification of H6 Subtype Avian Influenza Viruses,” Archives of Virology 163, no. 6 (2018): 1671–1675. 29468361 10.1007/s00705-018-3773-2

[77] C. C. Yip , W. M. Chan , J. D. Ip , et al., “Nanopore Sequencing Reveals Novel Targets for Detection and Surveillance of Human and Avian Influenza A Viruses,” Journal of Clinical Microbiology 58, no. 5 (2020): e02127‐19. 32132187 10.1128/JCM.02127-19 PMC7180252

[78] R. Parvin , C. K. Kabiraj , I. Hossain , et al., “Investigation of Respiratory Disease Outbreaks of Poultry in Bangladesh Using Two Real‐Time PCR‐Based Simultaneous Detection Assays,” Frontiers in Veterinary Science 9 (2022): 1036757. 36583036 10.3389/fvets.2022.1036757 PMC9792859

[79] S. Saito , I. Takayama , M. Nakauchi , et al., “Development and Evaluation of a New Real‐Time RT‐PCR Assay for Detecting the Latest H9N2 Influenza Viruses Capable of Causing Human Infection,” Microbiology and Immunology 63, no. 1 (2019): 21–31. 30599081 10.1111/1348-0421.12666 PMC6590187

[80] R. Zhang , D. Yao , J. Chen , et al., “Development and Evaluation of a Real‐Time RT‐PCR Assay for Detection of a Novel Avian Influenza A (H5N6) Virus,” Journal of Virological Methods 257 (2018): 79–84. 29729298 10.1016/j.jviromet.2018.05.001

[81] S. Saito , M. Nakauchi , I. Takayama , S. Nagata , T. Odagiri , and T. Kageyama , “Development and Evaluation of New Real‐Time RT‐PCR Assays for Identifying the Influenza A Virus Cluster IV H3N2 Variant,” Japanese Journal of Infectious Diseases 72, no. 2 (2019): 127–129. 30381693 10.7883/yoken.JJID.2018.395

[82] S. Azeem , B. Guo , D. Sun , M. L. Killian , J. A. Baroch , and K. J. Yoon , “Evaluation of PCR‐Based Hemagglutinin Subtyping as a Tool to Aid in Surveillance of Avian Influenza Viruses in Migratory Wild Birds,” Journal of Virological Methods 308 (2022): 114594. 35931229 10.1016/j.jviromet.2022.114594

[83] V. Haach , D. Gava , M. E. Cantao , and R. Schaefer , “Evaluation of Two Multiplex RT‐PCR Assays for Detection and Subtype Differentiation of Brazilian Swine Influenza Viruses,” Brazilian Journal of Microbiology 51, no. 3 (2020): 1447–1451. 32125678 10.1007/s42770-020-00250-z PMC7455636

[84] Z. Zhang , D. Liu , J. Hu , et al., “Multiplex One‐Step Real‐Time PCR Assay for Rapid Simultaneous Detection of Velogenic and Mesogenic Newcastle Disease Virus and H5‐Subtype Avian Influenza Virus,” Archives of Virology 164, no. 4 (2019): 1111–1119. 30790106 10.1007/s00705-019-04180-6

[85] M. Li , Z. Xie , Z. Xie , et al., “Simultaneous Detection of Eight Avian Influenza A Virus Subtypes by Multiplex Reverse Transcription‐PCR Using a GeXP Analyser,” Scientific Reports 8, no. 1 (2018): 6183. 29670227 10.1038/s41598-018-24620-8 PMC5906657

[86] V. Haach , D. Gava , E. C. Mauricio , A. C. Franco , and R. Schaefer , “One‐Step Multiplex RT‐qPCR for the Detection and Subtyping of Influenza A Virus in Swine in Brazil,” Journal of Virological Methods 269 (2019): 43–48. 30959063 10.1016/j.jviromet.2019.04.005

[87] N. B. Goecke , J. S. Krog , C. K. Hjulsager , et al., “Subtyping of Swine Influenza Viruses Using a High‐Throughput Real‐Time PCR Platform,” Frontiers in Cellular and Infection Microbiology 8 (2018): 165. 29872645 10.3389/fcimb.2018.00165 PMC5972299

[88] Z. Zhang , D. Liu , W. Sun , et al., “Multiplex One‐Step Real‐Time PCR by Taqman‐MGB Method for Rapid Detection of Pan and H5 Subtype Avian Influenza Viruses,” PLoS ONE 12, no. 6 (2017): e0178634. 28575115 10.1371/journal.pone.0178634 PMC5456101

[89] V. Panzarin , S. Marciano , A. Fortin , et al., “Redesign and Validation of a Real‐Time RT‐PCR to Improve Surveillance for Avian Influenza Viruses of the H9 Subtype,” Viruses 14, no. 6 (2022): 1263. 35746734 10.3390/v14061263 PMC9227555

[90] E. Bonin , S. Queguiner , C. Woudstra , et al., “Molecular Subtyping of European Swine Influenza Viruses and Scaling to High‐Throughput Analysis,” Virology Journal 15, no. 1 (2018): 7. 29316958 10.1186/s12985-018-0920-z PMC5761149

[91] Y. Li , L. Ni , J. Chen , J. Yang , F. Deng , and H. Wang , “Development of Multi‐Analyte Suspension Assay for Simultaneously Efficient Detection of Avian Influenza Virus A Subtypes,” Virologica Sinica 33, no. 1 (2018): 111–115. 29500693 10.1007/s12250-018-0018-1 PMC6178083

[92] H. Cui , C. Zhang , F. Tu , et al., “Rapid Detection of Influenza A Viruses Using a Real‐Time Reverse Transcription Recombinase‐Aided Amplification Assay,” Frontiers in Cellular and Infection Microbiology 12 (2022): 1071288. 36683681 10.3389/fcimb.2022.1071288 PMC9849684

[93] X. Zhou , S. Wang , Y. Ma , et al., “Rapid detection of Avian Influenza Virus Based on CRISPR‐Cas12a,” Virology Journal 20, no. 1 (2023): 261. 37957729 10.1186/s12985-023-02232-7 PMC10644463

[94] L. Xu , X. Jiang , Y. Zhu , et al., “A Multiplex Asymmetric Reverse Transcription‐PCR Assay Combined With an Electrochemical DNA Sensor for Simultaneously Detecting and Subtyping Influenza A Viruses,” Frontiers in Microbiology 9 (2018): 1405. 30013525 10.3389/fmicb.2018.01405 PMC6036258

[95] F. Cong , Y. Zhu , X. Liu , et al., “Development of an xTAG‐Multiplex PCR Array for the Detection of Four Avian Respiratory Viruses,” Molecular and Cellular Probes 37 (2018): 1–5. 29054443 10.1016/j.mcp.2017.10.002

[96] N. Liu , L. Wang , G. Cai , D. Zhang , and J. Lin , “Establishment of a Simultaneous Detection Method for Ten Duck Viruses Using MALDI‐TOF Mass Spectrometry,” Journal of Virological Methods 273 (2019): 113723. 31430495 10.1016/j.jviromet.2019.113723 PMC7113782

[97] K. Inui , T. Nguyen , H. J. Tseng , et al., “A Field‐Deployable Insulated Isothermal RT‐PCR Assay for Identification of Influenza A (H7N9) Shows Good Performance in the Laboratory,” Influenza and Other Respiratory Viruses 13, no. 6 (2019): 610–617. 31487118 10.1111/irv.12646 PMC6800302

[98] C. Guinat , D. Tago , T. Corre , et al., “Optimizing the Early Detection of Low Pathogenic Avian Influenza H7N9 Virus in Live Bird Markets,” Journal of the Royal Society Interface 18, no. 178 (2021): 20210074. 33947269 10.1098/rsif.2021.0074 PMC8097223

[99] J. Lee , A. Y. Cho , H. H. Ko , et al., “Evaluation of Insulated Isothermal PCR Devices for the Detection of Avian Influenza Virus,” Journal of Virological Methods 292 (2021): 114126. 33711374 10.1016/j.jviromet.2021.114126

[100] A. A. Bakre , L. P. Jones , H. K. Bennett , D. E. Bobbitt , and R. A. Tripp , “Detection of Swine Influenza Virus in Nasal Specimens by Reverse Transcription‐Loop‐Mediated Isothermal Amplification (RT‐LAMP),” Journal of Virological Methods 288 (2021): 114015. 33271254 10.1016/j.jviromet.2020.114015 PMC7799534

[101] S. Zhang , J. Shin , S. Shin , and Y. J. Chung , “Development of Reverse Transcription Loop‐Mediated Isothermal Amplification Assays for Point‐of‐Care Testing of Avian Influenza Virus Subtype H5 and H9,” Genomics & Informatics 18, no. 4 (2020): e40. 33412756 10.5808/GI.2020.18.4.e40 PMC7808867

[102] S. M. Storms , J. Shisler , T. H. Nguyen , F. A. Zuckermann , and J. F. Lowe , “RT‐LAMP as Diagnostic Tool for Influenza‐A Virus Detection in Swine,” Veterinary Sciences 10, no. 3 (2023): 220. 36977259 10.3390/vetsci10030220 PMC10051247

[103] S. J. Ahn , Y. H. Baek , K. K. S. Lloren , et al., “Rapid and Simple Colorimetric Detection of Multiple Influenza Viruses Infecting Humans Using a Reverse Transcriptional Loop‐Mediated Isothermal Amplification (RT‐LAMP) Diagnostic Platform,” BMC Infectious Diseases 19, no. 1 (2019): 676. 31370782 10.1186/s12879-019-4277-8 PMC6669974

[104] F. Filaire , A. Secula , L. Lebre , G. Croville , and J. L. Guerin , “A Real‐Time Colourimetric Reverse Transcription Loop‐Mediated Isothermal Amplification (RT‐LAMP) Assay for the Rapid Detection of Highly Pathogenic H5 Clade 2.3.4.4b Avian Influenza Viruses,” Avian Pathology 53, no. 2 (2024): 93–100. 37885409 10.1080/03079457.2023.2276849

[105] M. Golabi , M. Flodrops , B. Grasland , et al., “Development of Reverse Transcription Loop‐Mediated Isothermal Amplification Assay for Rapid and On‐Site Detection of Avian Influenza Virus,” Frontiers in Cellular and Infection Microbiology 11 (2021): 652048. 33954120 10.3389/fcimb.2021.652048 PMC8092359

[106] Y. Yao , X. Chen , X. Zhang , et al., “Rapid Detection of Influenza Virus Subtypes Based on an Integrated Centrifugal Disc,” ACS Sensors 5, no. 5 (2020): 1354–1362. 32248677 10.1021/acssensors.9b02595

[107] J. Park , J. Kim , C. Park , et al., “A Flap Endonuclease 1‐Assisted Universal Viral Nucleic Acid Sensing System Using Surface‐Enhanced Raman Scattering,” Analyst 147, no. 22 (2022): 5028–5037. 36190457 10.1039/d2an01123a

[108] Y. Sun , L. Xu , F. Zhang , et al., “A Promising Magnetic SERS Immunosensor for Sensitive Detection of Avian Influenza Virus,” Biosensors & Bioelectronics 89, no. Pt 2 (2017): 906–912. 27818055 10.1016/j.bios.2016.09.100

[109] M. Xiao , K. Xie , X. Dong , et al., “Ultrasensitive Detection of Avian Influenza A (H7N9) Virus Using Surface‐Enhanced Raman Scattering‐Based Lateral Flow Immunoassay Strips,” Analytica Chimica Acta 1053 (2019): 139–147. 30712559 10.1016/j.aca.2018.11.056

[110] B. T. Duong , D. D. Than , B. G. Ju , et al., “Development of a Rapid Fluorescent Diagnostic System for Early Detection of the Highly Pathogenic Avian Influenza H5 Clade 2.3.4.4 Viruses in Chicken Stool,” International Journal of Molecular Sciences 23, no. 11 (2022): 6301. 35682982 10.3390/ijms23116301 PMC9181406

[111] H. T. Tuong , J. H. Jeong , Y. K. Choi , H. Park , Y. H. Baek , and S. J. Yeo , “Development of a Rapid Fluorescent Diagnostic System to Detect Subtype H9 Influenza A Virus in Chicken Feces,” International Journal of Molecular Sciences 22, no. 16 (2021): 8823. 34445529 10.3390/ijms22168823 PMC8396311

[112] J. Huang , Z. Xie , L. Xie , et al., “Au/Fe(3)O(4) Core‐Shell Nanoparticles Are an Efficient Immunochromatography Test Strip Performance Enhancer—A Comparative Study With Au and Fe(3)O(4) Nanoparticles,” RSC Advances 8, no. 25 (2018): 14064–14071. 35539327 10.1039/c8ra00185e PMC9079878

[113] L. T. Nguyen , K. Nakaishi , K. Motojima , et al., “Rapid and Broad Detection of H5 Hemagglutinin by an Immunochromatographic Kit Using Novel Monoclonal Antibody Against Highly Pathogenic Avian Influenza Virus Belonging to the Genetic Clade 2.3.4.4,” PLoS ONE 12, no. 8 (2017): e0182228. 28787440 10.1371/journal.pone.0182228 PMC5546692

[114] K. Iwatsuki‐Horimoto , J. Shi , X. Wang , et al., “Development of an Influenza Rapid Diagnostic Kit Specific for the H7 Subtype,” Frontiers in Microbiology 9 (2018): 1346. 29988537 10.3389/fmicb.2018.01346 PMC6026626

[115] F. Yang , Y. Xiao , B. Chen , et al., “Development of a Colloidal Gold‐Based Immunochromatographic Strip Test Using Two Monoclonal Antibodies to Detect H7N9 Avian Influenza Virus,” Virus Genes 56, no. 3 (2020): 396–400. 32034616 10.1007/s11262-020-01742-8

[116] M. Xiao , L. Huang , X. Dong , et al., “Integration of a 3D‐Printed Read‐Out Platform With a Quantum Dot‐Based Immunoassay for Detection of the Avian Influenza A (H7N9) Virus,” Analyst 144, no. 8 (2019): 2594–2603. 30830133 10.1039/c8an02336k

[117] G. Li , X. Wang , Q. Li , et al., “Development of an Immunochromatographic Strip for Rapid Detection of H7 Subtype Avian Influenza Viruses,” Virology Journal 18, no. 1 (2021): 68. 33827632 10.1186/s12985-021-01537-9 PMC8025375

[118] Y. Zhang , F. Mu , Y. Duan , et al., “Label‐Free Analysis of H5N1 Virus Based on Three‐Segment Branched DNA‐Templated Fluorescent Silver Nanoclusters,” ACS Applied Materials & Interfaces 12, no. 43 (2020): 48357–48362. 33052659 10.1021/acsami.0c14509

[119] X. Yu , Y. Xia , Y. Tang , et al., “A Nanostructured Microfluidic Immunoassay Platform for Highly Sensitive Infectious Pathogen Detection,” Small 13, no. 24 (2017): 1700425. 28636164 10.1002/smll.201700425 PMC7169616

[120] D. T. Bao , D. T. H. Kim , H. Park , et al., “Rapid Detection of Avian Influenza Virus by Fluorescent Diagnostic Assay using an Epitope‐Derived Peptide,” Theranostics 7, no. 7 (2017): 1835–1846. 28638471 10.7150/thno.18857 PMC5479272

[121] D. Jiang , Y. Tian , Y. Zhang , X. Lu , D. Xiao , and C. Zhou , “One‐Step Fast and Label‐Free Imaging Array for Multiplexed Detection of Trace Avian Influenza Viruses,” Analytica Chimica Acta 1171 (2021): 338645. 34112438 10.1016/j.aca.2021.338645

[122] L. Y. Hung , J. C. Chang , Y. C. Tsai , et al., “Magnetic Nanoparticle‐Based Immunoassay for Rapid Detection of Influenza Infections by Using an Integrated Microfluidic System,” Nanomedicine: Nanotechnology, Biology and Medicine 10, no. 4 (2014): 819–829. 24333595 10.1016/j.nano.2013.11.009 PMC7106285

[123] W. Wei‐Wen Hsiao , G. Fadhilah , C. C. Lee , et al., “Nanomaterial‐Based Biosensors for Avian Influenza Virus: A New Way Forward,” Talanta 265 (2023): 124892. 37451119 10.1016/j.talanta.2023.124892

[124] S. J. Yeo , D. T. Bao , G. E. Seo , et al., “Improvement of a Rapid Diagnostic Application of Monoclonal Antibodies Against Avian Influenza H7 Subtype Virus Using Europium Nanoparticles,” Scientific Reports 7, no. 1 (2017): 7933. 28801679 10.1038/s41598-017-08328-9 PMC5554140

[125] G. Park , J. W. Lim , C. Park , et al., “Cell‐Mimetic Biosensors to Detect Avian Influenza Virus via Viral Fusion,” Biosensors & Bioelectronics 212 (2022): 114407. 35623252 10.1016/j.bios.2022.114407

[126] K. Durairaj , D. D. Than , A. T. V. Nguyen , H. S. Kim , S. J. Yeo , and H. Park , “Cysteamine‐Gold Coated Carboxylated Fluorescent Nanoparticle Mediated Point‐of‐Care Dual‐Modality Detection of the H5N1 Pathogenic Virus,” International Journal of Molecular Sciences 23, no. 14 (2022): 7957. 35887315 10.3390/ijms23147957 PMC9320457

[127] L. Zheng , J. Wei , X. Lv , et al., “Detection and Differentiation of Influenza Viruses With Glycan‐Functionalized Gold Nanoparticles,” Biosensors & Bioelectronics 91 (2017): 46–52. 27987410 10.1016/j.bios.2016.12.037

[128] N. Kumar , S. Bhatia , A. K. Pateriya , et al., “Label‐Free Peptide Nucleic Acid Biosensor for Visual Detection of Multiple Strains of Influenza A Virus Suitable for Field Applications,” Analytica Chimica Acta 1093 (2020): 123–130. 31735205 10.1016/j.aca.2019.09.060

[129] J. Kim , J. H. Kwon , J. Jang , et al., “Rapid and Background‐Free Detection of Avian Influenza Virus in Opaque Sample Using NIR‐to‐NIR Upconversion Nanoparticle‐Based Lateral Flow Immunoassay Platform,” Biosensors & Bioelectronics 112 (2018): 209–215. 29709831 10.1016/j.bios.2018.04.047

[130] Q. Zhang , G. Rawal , J. Qian , et al., “An Integrated Magneto‐Opto‐Fluidic Biosensor for Rapid On‐Chip Assay of Respiratory Viruses of Livestock,” Lab on a Chip 22, no. 17 (2022): 3236–3244. 35875988 10.1039/d2lc00406b

[131] L. Chen , F. Ruan , M. Liu , J. Zhou , W. Song , and K. Qin , “A Sandwich ELISA for Detecting the Hemagglutinin of Avian Influenza A (H10N8) Virus,” Journal of Medical Virology 91, no. 5 (2019): 877–880. 30593681 10.1002/jmv.25387

[132] Y. Xiao , F. Yang , F. Liu , H. Yao , N. Wu , and H. Wu , “Antigen‐Capture ELISA and Immunochromatographic Test Strip to Detect the H9N2 Subtype Avian Influenza Virus Rapidly Based on Monoclonal Antibodies,” Virology Journal 18, no. 1 (2021): 198. 34600550 10.1186/s12985-021-01671-4 PMC8487345

[133] F. Ming , Y. Cheng , C. Ren , S. Suolang , and H. Zhou , “Development of a DAS‐ELISA for Detection of H9N2 Avian Influenza Virus,” Journal of Virological Methods 263 (2019): 38–43. 30355516 10.1016/j.jviromet.2018.10.014

[134] S. Luo , X. Deng , Z. Xie , et al., “Production and Identification of Monoclonal Antibodies and Development of a Sandwich ELISA for Detection of the H3‐Subtype Avian Influenza Virus Antigen,” AMB Express 10, no. 1 (2020): 49. 32170425 10.1186/s13568-020-00988-7 PMC7070111

[135] Y. Yu , Z. Zhang , H. Li , et al., “Biological Characterizations of H5Nx Avian Influenza Viruses Embodying Different Neuraminidases,” Frontiers in Microbiology 8 (2017): 1084. 28659898 10.3389/fmicb.2017.01084 PMC5469879

[136] C. Aira , J. I. Klett‐Mingo , T. Ruiz , et al., “Development of an Antigen Enzyme‐Linked AptaSorbent Assay (ELASA) for the Detection of Swine Influenza Virus in Field Samples,” Analytica Chimica Acta 1181 (2021): 338933. 34556218 10.1016/j.aca.2021.338933

[137] F. Yang , Y. Xiao , L. Xu , et al., “Development of an Antigen‐Capture Enzyme‐Linked Immunosorbent Assay and Immunochromatographic Strip Based on Monoclonal Antibodies for Detection of H6 Avian Influenza Viruses,” Archives of Virology 165, no. 5 (2020): 1129–1139. 32221715 10.1007/s00705-020-04602-w

[138] D. Sila‐On , P. Chertchinnapa , Y. Shinkai , T. Kojima , and H. Nakano , “Development of a Dual Monoclonal Antibody Sandwich Enzyme‐Linked Immunosorbent Assay for the Detection of Swine Influenza Virus Using Rabbit Monoclonal Antibody by Ecobody Technology,” Journal of Bioscience and Bioengineering 130, no. 2 (2020): 217–225. 32284304 10.1016/j.jbiosc.2020.03.003

[139] F. Yang , Y. Xiao , F. Liu , H. Yao , N. Wu , and H. Wu , “Development of a Monoclonal Antibody‐Based Antigen Capture Enzyme‐Linked Immunosorbent Assay for Detection of H7N9 Subtype Avian Influenza Virus,” Journal of Medical Virology 93, no. 6 (2021): 3939–3943. 32648948 10.1002/jmv.26292

[140] H. Takahashi , S. Nagata , T. Odagiri , and T. Kageyama , “Establishment of the Cross‐Clade Antigen Detection System for H5 Subtype Influenza Viruses Using Peptide Monoclonal Antibodies Specific for Influenza Virus H5 Hemagglutinin,” Biochemical and Biophysical Research Communications 498, no. 4 (2018): 758–763. 29524417 10.1016/j.bbrc.2018.03.054

[141] S. H. Kwon , S. Lee , J. Jang , Y. Seo , and H. Y. Lim , “A Point‐of‐Care Diagnostic System to Influenza Viruses Using Chip‐Based Ultra‐Fast PCR,” Journal of Medical Virology 90, no. 6 (2018): 1019–1026. 29424457 10.1002/jmv.25046

[142] D. Su , K. Wu , V. D. Krishna , et al., “Detection of Influenza a Virus in Swine Nasal Swab Samples With a Wash‐Free Magnetic Bioassay and a Handheld Giant Magnetoresistance Sensing System,” Frontiers in Microbiology 10 (2019): 1077. 31164877 10.3389/fmicb.2019.01077 PMC6536586

[143] H. Jung , S. H. Park , J. Lee , et al., “A Size‐Selectively Biomolecule‐Immobilized Nanoprobe‐Based Chemiluminescent Lateral Flow Immunoassay for Detection of Avian‐Origin Viruses,” Analytical Chemistry 93, no. 2 (2021): 792–800. 33175513 10.1021/acs.analchem.0c03153

[144] Y. Xiao , J. M. Nolting , Z. M. Sheng , et al., “Design and Validation of a Universal Influenza Virus Enrichment Probe Set and Its Utility in Deep Sequence Analysis of Primary Cloacal Swab Surveillance Samples of Wild Birds,” Virology 524 (2018): 182–191. 30212665 10.1016/j.virol.2018.08.021 PMC6617512

[145] S. J. Yeo , B. T. Cuc , S. A. Kim , et al., “Rapid Detection of Avian Influenza A Virus by Immunochromatographic Test Using a Novel Fluorescent Dye,” Biosensors & Bioelectronics 94 (2017): 677–685. 28390319 10.1016/j.bios.2017.03.068

[146] J. Yan , Q. Cheng , H. Liu , L. Wang , and K. Yu , “Sensitive and Rapid Detection of Influenza A Virus for Disease Surveillance Using Dual‐Probe Electrochemical Biosensor,” Bioelectrochemistry 153 (2023): 108497. 37393678 10.1016/j.bioelechem.2023.108497

[147] K. S. Kuchinski , J. Duan , C. Himsworth , W. Hsiao , and N. A. Prystajecky , “ProbeTools: Designing Hybridization Probes for Targeted Genomic Sequencing of Diverse and Hypervariable Viral Taxa,” BMC Genomics 23, no. 1 (2022): 579. 35953803 10.1186/s12864-022-08790-4 PMC9371634

[148] G. Yang , E. N. Hodges , J. Winter , et al., “Development of an RNA Strand‐Specific Hybridization Assay to Differentiate Replicating Cersus Nonreplicating Influenza A Viruses,” Journal of Clinical Microbiology 58, no. 6 (2020): e00252‐20. 32245834 10.1128/JCM.00252-20 PMC7269401

[149] X. Peng , G. Luo , Z. Wu , W. Wen , X. Zhang , and S. Wang , “Fluorescent‐Magnetic‐Catalytic Nanospheres for Dual‐Modality Detection of H9N2 Avian Influenza Virus,” ACS Applied Materials & Interfaces 11, no. 44 (2019): 41148–41156. 31613583 10.1021/acsami.9b16718

[150] A. Sampieri , R. Monroy‐Contreras , A. Asanov , and L. Vaca , “Design of Hydrogel Silk‐Based Microarrays and Molecular Beacons for Reagentless Point‐of‐Care Diagnostics,” Frontiers in Bioengineering and Biotechnology 10 (2022): 881679. 35957640 10.3389/fbioe.2022.881679 PMC9361048

[151] H. Xiang , X. Wen , Y. Wen , et al., “Development and Application of a Visual Microarray for Synchronously Detecting H5N1, H7N9 and H9N2 Avian Influenza Virus RNA,” Journal of Virological Methods 301 (2022): 114371. 34808230 10.1016/j.jviromet.2021.114371

[152] H. Zhang , A. M. Klose , and B. L. Miller , “Label‐Free, Multiplex Glycan Microarray Biosensor for Influenza Virus Detection,” Bioconjugate Chemistry 32, no. 3 (2021): 533–540. 33559468 10.1021/acs.bioconjchem.0c00718

[153] Q. Xiao , L. Yan , L. Yao , et al., “Development of Oligonucleotide Microarray for Accurate and Simultaneous Detection of Avian Respiratory Viral Diseases,” BMC Veterinary Research 15, no. 1 (2019): 253. 31324180 10.1186/s12917-019-1985-7 PMC6642548

[154] K. T. Sultankulova , N. S. Kozhabergenov , V. M. Strochkov , et al., “New Oligonucleotide Microarray for Rapid Diagnosis of Avian Viral Diseases,” Virology Journal 14, no. 1 (2017): 69. 28381285 10.1186/s12985-017-0738-0 PMC5382490

[155] H. Zhang , C. Henry , C. S. Anderson , et al., “Crowd on a Chip: Label‐Free Human Monoclonal Antibody Arrays for Serotyping Influenza,” Analytical Chemistry 90, no. 15 (2018): 9583–9590. 29985597 10.1021/acs.analchem.8b02479 PMC6082710

[156] T. Matsubara , M. Ujie , T. Yamamoto , et al., “Avian Influenza Virus Detection by Optimized Peptide Termination on a Boron‐Doped Diamond Electrode,” ACS Sensors 5, no. 2 (2020): 431–439. 32077684 10.1021/acssensors.9b02126

[157] D. Lee , J. Bhardwaj , and J. Jang , “Paper‐Based Electrochemical Immunosensor for Label‐Free Detection of Multiple Avian Influenza Virus Antigens Using Flexible Screen‐Printed Carbon Nanotube‐Polydimethylsiloxane Electrodes,” Scientific Reports 12, no. 1 (2022): 2311. 35145121 10.1038/s41598-022-06101-1 PMC8831593

[158] J. Huang , Z. Xie , M. Li , et al., “An Enzyme‐Free Sandwich Amperometry‐Type Immunosensor Based on Au/Pt Nanoparticle‐Functionalized Graphene for the Rapid Detection of Avian Influenza Virus H9 Subtype,” Nanoscale Research Letters 17, no. 1 (2022): 110. 36404373 10.1186/s11671-022-03747-8 PMC9676155

[159] B. A.‐O. Luo , Z. Liu , X. Wang , et al., “Dual‐Peak Long Period Fiber Grating Coated With Graphene Oxide for Label‐Free and Specific Assays of H5N1 Virus,” Journal of Biophotonics 14, no. 1 (2021): e202000279. 32902141 10.1002/jbio.202000279

[160] A. Roberts , N. Chauhan , S. Islam , et al., “Graphene Functionalized Field‐Effect Transistors for Ultrasensitive Detection of Japanese Encephalitis and Avian Influenza Virus,” Scientific Reports 10, no. 1 (2020): 14546. 32884083 10.1038/s41598-020-71591-w PMC7471952

[161] J. King , T. Harder , M. Beer , and A. Pohlmann , “Rapid Multiplex MinION Nanopore Sequencing Workflow for Influenza A Viruses,” BMC Infectious Diseases 20, no. 1 (2020): 648. 32883215 10.1186/s12879-020-05367-y PMC7468549

[162] S. L. Butt , H. M. Kariithi , J. D. Volkening , et al., “Comparable Outcomes From Long and Short Read Random Sequencing of Total RNA for Detection of Pathogens in Chicken Respiratory Samples,” Frontiers in Veterinary Science 9 (2022): 1073919. 36532355 10.3389/fvets.2022.1073919 PMC9751482

[163] A. C. Neujahr , D. S. Loy , J. D. Loy , B. W. Brodersen , and S. C. Fernando , “Rapid Detection of High Consequence and Emerging Viral Pathogens in Pigs,” Frontiers in Veterinary Science 11 (2024): 1341783. 38384961 10.3389/fvets.2024.1341783 PMC10879307

[164] B. M. Crossley , D. Rejmanek , J. Baroch , et al., “Nanopore Sequencing as a Rapid Tool for Identification and Pathotyping of Avian Influenza A Viruses,” Journal of Veterinary Diagnostic Investigation 33, no. 2 (2021): 253–260. 33550926 10.1177/1040638720984114 PMC7953088

[165] L. Schuele , E. Lizarazo‐Forero , K. Strutzberg‐Minder , et al., “Application of Shotgun Metagenomics Sequencing and Targeted Sequence Capture to Detect Circulating Porcine Viruses in the Dutch‐German Border Region,” Transboundary and Emerging Diseases 69, no. 4 (2022): 2306–2319. 34347385 10.1111/tbed.14249 PMC9540031

[166] E. M. de Vries , N. O. I. Cogan , A. J. Gubala , et al., “Rapid, In‐Field Deployable, Avian Influenza Virus Haemagglutinin Characterisation Tool Using MinION Technology,” Scientific Reports 12, no. 1 (2022): 11886. 35831457 10.1038/s41598-022-16048-y PMC9279447

[167] K. Lewandowski , Y. Xu , S. T. Pullan , et al., “Metagenomic Nanopore Sequencing of Influenza Virus Direct from Clinical Respiratory Samples,” Journal of Clinical Microbiology 58, no. 1 (2019): e00963‐19. 31666364 10.1128/JCM.00963-19 PMC6935926

[168] T. T. Lam and O. G. Pybus , “Genomic Surveillance of Avian‐Origin Influenza A Viruses Causing Human Disease,” Genome Medicine 10, no. 1 (2018): 50. 29950176 10.1186/s13073-018-0560-3 PMC6020380

[169] Y. Qiu , S. Wang , B. Huang , et al., “Viral Infection Detection Using Metagenomics Technology in Six Poultry Farms of Eastern China,” PLoS ONE 14, no. 2 (2019): e0211553. 30785912 10.1371/journal.pone.0211553 PMC6382132

[170] L. Penarrubia , S. N. Rao , R. Porco , et al., “Detecting Zoonotic Influenza A using QIAstat‐Dx Respiratory SARS‐CoV‐2 Panel for Pandemic Preparedness,” Scientific Reports 13, no. 1 (2023): 2833. 36807577 10.1038/s41598-023-29838-9 PMC9936110

[171] G. Manessis , M. Frant , G. Wozniakowski , et al., “Point‐of‐Care and Label‐Free Detection of Porcine Reproductive and Respiratory Syndrome and Swine Influenza Viruses Using a Microfluidic Device with Photonic Integrated Circuits,” Viruses 14, no. 5 (2022): 988. 35632730 10.3390/v14050988 PMC9144544

[172] K. Wu , T. Klein , V. D. Krishna , D. Su , A. M. Perez , and J. P. Wang , “Portable GMR Handheld Platform for the Detection of Influenza A Virus,” ACS Sensors 2, no. 11 (2017): 1594–1601. 29068663 10.1021/acssensors.7b00432

[173] L. Jung Kjaer , M. P. Ward , A. E. Boklund , L. E. Larsen , C. K. Hjulsager , and C. T. Kirkeby , “Author Correction: Using Surveillance Data for Early Warning Modelling of Highly Pathogenic Avian Influenza in Europe Reveals a Seasonal Shift in Transmission, 2016‐2022,” Scientific Reports 13, no. 1 (2023): 16612. 37789088 10.1038/s41598-023-43740-4 PMC10547703

[174] WHO . What Research Is Important to Prepare and Respond to H5N1 Influenza Outbreaks? (2025), https://www.who.int/news‐room/events/detail/2025/03/19/default‐calendar/what‐research‐is‐important‐to‐prepare‐and‐respond‐to‐h5n1‐influenza‐outbreaks.

